# Optimal experimental designs for estimating genetic and non-genetic effects underlying infectious disease transmission

**DOI:** 10.1186/s12711-022-00747-1

**Published:** 2022-09-05

**Authors:** Christopher Pooley, Glenn Marion, Stephen Bishop, Andrea Doeschl-Wilson

**Affiliations:** 1 Biomathematics and Statistics Scotland, James Clerk Maxwell Building, The King’s Buildings, Peter Guthrie Tait Road, Edinburgh, EH9 3FD UK; 2 The Roslin Institute, University of Edinburgh, Midlothian, EH25 9RG UK

## Abstract

**Background:**

The spread of infectious diseases in populations is controlled by the susceptibility (propensity to acquire infection), infectivity (propensity to transmit infection), and recoverability (propensity to recover/die) of individuals. Estimating genetic risk factors for these three underlying host epidemiological traits can help reduce disease spread through genetic control strategies. Previous studies have identified important ‘disease resistance single nucleotide polymorphisms (SNPs)’, but how these affect the underlying traits is an unresolved question. Recent advances in computational statistics make it now possible to estimate the effects of SNPs on host traits from epidemic data (e.g. infection and/or recovery times of individuals or diagnostic test results). However, little is known about how to effectively design disease transmission experiments or field studies to maximise the precision with which these effects can be estimated.

**Results:**

In this paper, we develop and validate analytical expressions for the precision of the estimates of SNP effects on the three above host traits for a disease transmission experiment with one or more non-interacting contact groups. Maximising these expressions leads to three distinct ‘experimental’ designs, each specifying a different set of ideal SNP genotype compositions across groups: (a) appropriate for a single contact-group, (b) a multi-group design termed “pure”, and (c) a multi-group design termed “mixed”, where ‘pure’ and ‘mixed’ refer to groupings that consist of individuals with uniformly the same or different SNP genotypes, respectively. Precision estimates for susceptibility and recoverability were found to be less sensitive to the experimental design than estimates for infectivity. Whereas the analytical expressions suggest that the multi-group pure and mixed designs estimate SNP effects with similar precision, the mixed design is preferred because it uses information from naturally-occurring rather than artificial infections. The same design principles apply to estimates of the epidemiological impact of other categorical fixed effects, such as breed, line, family, sex, or vaccination status. Estimation of SNP effect precisions from a given experimental setup is implemented in an online software tool *SIRE-PC*.

**Conclusions:**

Methodology was developed to aid the design of disease transmission experiments for estimating the effect of individual SNPs and other categorical variables that underlie host susceptibility, infectivity and recoverability. Designs that maximize the precision of estimates were derived.

**Supplementary Information:**

The online version contains supplementary material available at 10.1186/s12711-022-00747-1.

## Background

Infectious disease constitutes one of the biggest threats to sustainable livestock and aquaculture production, global food security, and human health. Over the last decades, genome-wide association studies (GWAS), together with high-density sequencing and other ‘omics’ technologies, have facilitated enormous breakthroughs in disease genetics, with the number of genetic loci that have been identified to be associated with disease resistance increasing at a rapid rate [1–6]. Accordingly, expectations for reducing infectious disease prevalence through genetic selection for disease resistance are increasing, and some real-world applications have demonstrated that these expectations can be met in practice [7].

The most effective way to reduce infectious disease prevalence in a population is to reduce the individuals’ susceptibility to infection or their ability to transmit infections, once infected. Yet, remarkably little is known about the role of previously identified ‘resistance’ loci in infectious disease transmission, because in most studies, disease resistance refers to the resistance of an infected animal to develop disease or other side-effects from infection (e.g. performance reduction or death), rather than to resistance to becoming infected or transmitting the infection [8–10]. Hence, it is not known whether selection for disease resistance actually reduces disease prevalence, since animals that carry the beneficial resistance alleles may still become infected and transmit the infection. Furthermore, discovery of single nucleotide polymorphisms (SNPs) associated with disease resistance often originate from large-scale disease challenge experiments, in which individuals are artificially infected or exposed to a specific pathogen strain and dose, and their response to infection is measured [11–13]. However, estimating the effect of genetic loci identified in these studies on traits associated with disease transmission would require field or experimental epidemic data from situations where the infection is transmitted naturally between individuals.

Epidemiological models are widely used to identify risk factors for disease transmission in populations and to assess the impact of control measures on these. Particularly relevant for genetically heterogeneous populations are compartmental models, in which individuals are classified as, for example, susceptible to infection (S), infected and infectious (I), or recovered/removed (dead) (R) [14]. These epidemiological SIR models point naturally to three distinct host genetic traits that characterise the key processes of disease transmission dynamics within a population: individual *susceptibility*, *infectivity*, and *recoverability* [15–17]. In an epidemiological context, *susceptibility* is defined as the relative risk of an uninfected individual becoming infected when exposed to a typical infectious individual or to infectious material excreted from such an individual, *infectivity* is the propensity of an infected individual to transmit infection to a typical (average) susceptible individual, and *recoverability* is the propensity of an infected individual to recover or die [15, 18, 19]. For SIR models, recoverability is the inverse of the mean duration for the infectious period.

Conceptually, genetic improvement in any or all three of these underlying epidemiological host traits is expected to reduce disease spread within and across populations. Indeed, recent advances in treating infection partly as an indirect genetic effect (IGE) have pointed to far greater responses to selection than had previously been expected [20, 21]. This has been demonstrated for infectious pancreatic necrosis (IPN), a viral disease that inflicts high mortality in Atlantic salmon populations. Previous GWAS had identified a single quantitative trait locus (QTL) that explains over 80% of the genetic variation in mortality caused by IPN [22, 23]. The corresponding candidate gene that was identified in subsequent fine-mapping studies was found to primarily control IPN virus internalization, i.e. host susceptibility [24]. A small-scale IPN transmission experiment, in which fish were assigned into different epidemic groups according to their QTL genotypes, provided evidence that the beneficial allele reduced the infectivity of IPN-infected fish, in addition to reducing their susceptibility, and may also have favourable effects on duration of the infectious period (i.e. their recoverability) [25]. This beneficial pleiotropic effect on all three epidemiological host traits may explain why breeding schemes for IPN-resistance have led to a drastic reduction in IPN prevalence and associated mortalities within just a few generations of selection [26]. Incorporating host traits into epidemiological models can also inform management strategies on how to effectively prevent disease outbreaks in genetically heterogeneous populations [25]. In this case, the aim is to reduce the basic reproduction number to less to 1, which can be achieved in fewer generations if multiple traits are targeted, e.g. susceptibility and infectivity, rather than just susceptibility [27] or resistance.

Compared to conventional disease resistance traits used in most GWAS (e.g*.* infection, disease, or survival status, or measures of pathogen load, immune response, or performance after infection challenge), the three epidemiological host traits have the clear advantage that their role in disease spread is fully specified by epidemiological models. However, until recently, estimation of genetic effects for these traits has proven challenging, as they need to be inferred from observable disease phenotypes. Fortunately, recent advances in computational statistics now enable genetic effects for host traits to be estimated from longitudinal disease records of individuals [15, 28–31]. However, how disease transmission experiments should be designed to obtain accurate estimates has received little attention. For example, previous studies have indicated that accurate estimation of genetic infectivity effects requires genetically-related individuals that are distributed across different contact groups [15, 28, 29] and that relatedness among group members can substantially affect precision and bias of the effect estimates [29, 32]. Hence, the effects of the number and size of contact groups, the genetic composition of individuals within groups, and of other parameters on precision of estimates need to be established.

In reality, there may be many SNPs that each have different effects on susceptibility, infectivity, and recovery, with possible epistatic interactions. However, in practice disentangling these interactions is usually not possible, both computationally and practically. Thus, this paper focuses on designs to determine the effect of a single SNP that, e.g. based on previous studies, is known to have a large effect on a resistance phenotype on the three epidemiological host traits (with multiple analyses performed in the case of several such SNPs). Our objectives were: (1) to derive analytical expressions for the precision of estimates of the effects of a SNP on the three underlying epidemiological host traits; for tractability, these expressions assume a best-case scenario (i.e. infection and recovery times are exactly known and other potentially confounding factors are ignored) and, therefore, represent upper bounds for precision of estimates from real data; (2) to use these insights to develop optimal designs of disease transmission experiments that aim at estimating the effects of a single SNP of interest on host susceptibility, infectivity, and recoverability; (3) to validate the analytical expressions and designs for a range of realistic data scenarios, e.g. the inclusion of group effects, other fixed effects, and residual noise, and cases when only the deaths of individuals are recorded and infection times are unknown; and (4) to present an easy-to-use online software tool to assist in the construction of a suitable design for a disease transmission experiment.

Although this study focuses on the estimation of SNP effects underlying disease transmission, the developed methodology and optimal design principles also apply to investigating the effects of other categorical variables (such as breed, line, family, sex, vaccination status,^1^ etc.) on host susceptibility, infectivity, and recoverability. Additional information on the application and extension of the developed methods and results presented here to identifying loci associated with disease transmission in a GWAS and application to field data are described in the “Discussion” section.

## Methods

### Key concepts, assumptions, terminology and data

**Fig. 1:**
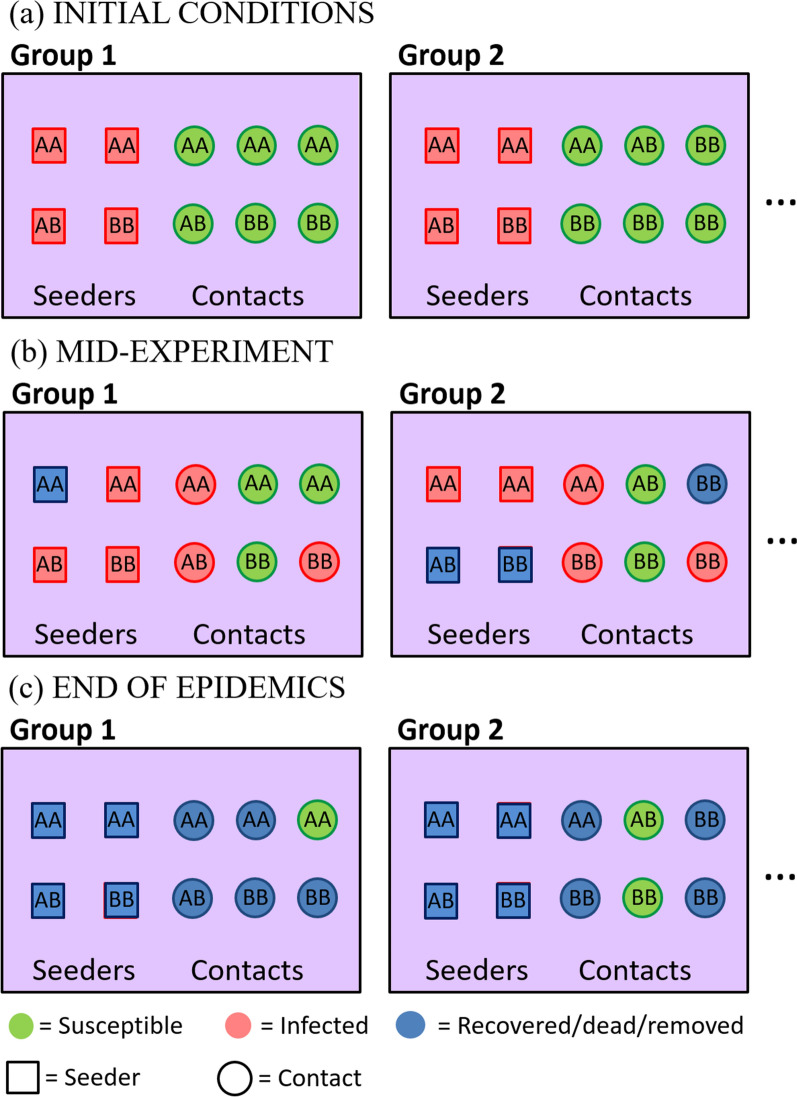
Schematic diagram of a disease transmission experiment. **a** The experiment consists of several contact groups in which some individuals are initially infected “seeders” and some are initially susceptible “contacts”. Each symbol represents an individual, and the annotations *AA*, *AB* and *BB* refer to the genotype of that individual at a given bi-allelic SNP under investigation. **b** As the experiment progresses some susceptible individuals become infected and some infected individuals recover. **c** If the experiment continues until the epidemics die out, only susceptible and recovered individuals are observed in the final state (for practical reasons, experiments are often terminated before this point). Note that the spatial separation of seeders (left) and contacts (right) in this diagram is for illustrative purposes only (random mixing between individuals is assumed)

To introduce the terminology and assumptions made in this study, Fig. 1 illustrates the key features of a disease transmission experiment in farmed animals. The experiment typically consists of one or more “contact groups”, where a “contact” is defined as being any interaction that allows for a disease to be transmitted from one individual to another (e.g*.* physical contact, via aerosol transmission, or contamination of the environment^2^). Importantly, contacts are assumed to occur randomly within groups but no contacts (and hence no transmission) occurs between groups.

In this study, which focuses on estimation of the effects of a particular SNP, it is assumed that individuals are randomly distributed across contact groups with regards to genetic effects on the epidemiological traits that are not captured by the SNP under consideration (see “Discussion”). This implies, for example, that related individuals (e.g. full-sibs or half-sibs) are assumed to be equally distributed across contact groups. The overall population is assumed to be composed of diploid individuals with a bi-allelic genetic structure, such that “*A*” and “*B*” represent different alleles at the SNP or genetic locus under investigation, resulting in three potential genotypes {*AA,AB,BB*} (Fig. 1).

The transmission experiment starts with two types of individuals (Fig. 1a): “seeders”, which are infected (either artificially or from prior exposure to other infected individuals^3^) at the beginning of the experiment, and “contacts”, which are susceptible to the disease. After some time (Fig. 1b) the infection has passed from the seeders to some of the contacts, and possibly also between contacts, while some infected individuals may have recovered from disease. Note, “recovered” can also refer to an individual which has died, and the two are used here synonymously.^4^ Eventually (Fig. 1c), all infected individuals have recovered and typically some susceptible individuals remain that did not become infected. In reality, the experiment may be terminated before all epidemics have finished, in which case censoring of the data will need to be accounted for in the analysis. In this study, it is assumed that transmission dynamics are the same for seeder-to-contact individuals as for contact-to-contact individuals (in reality this may not be the case, and this has important implications for optimal experimental design, as highlighted in the “Discussion” section later).

For each group the genotypic makeup for the target SNP is characterised by the following key quantities: ${H}_{\mathrm{seed}}$ and ${H}_{\mathrm{cont}}$ represent the proportion of homozygotes (i.e.* AA* or *BB*) in the seeders and contacts, respectively, and ${\chi }_{\mathrm{seed}}$ and ${\chi }_{\mathrm{cont}}$ represent the so-called “homozygote balance”, defined as the proportion of *AA* minus the proportion of *BB* individuals.^5^ Together, ${H}_{\mathrm{seed}}$, ${\chi }_{\mathrm{seed}}$, ${H}_{\mathrm{cont}}$ and ${\chi }_{\mathrm{cont}}$ define the three genotype frequencies in the seeder and contact populations that are controlled by the researcher (such that deviations from Hardy–Weinberg equilibrium may be created on purpose). These quantities are used in later analysis and are summarised in Table 1, along with other key parameters (described below).

**Table 1:** List of key parameters and quantities

Type	Parameter	Description
Experimental design	${N}_{\mathrm{group}}$	Number of contact groups
	${N}_{\mathrm{seed}}$	Number of seeders (initially infected individuals) in each contact group
	${N}_{\mathrm{cont}}$	Number of contacts (initially susceptible individuals) in each contact group
	${G}_{\mathrm{size}}$	Total number of individuals per group ${G}_{\mathrm{size}}={N}_{\mathrm{seed}}+{N}_{\mathrm{cont}}$
	${N}_{\mathrm{total}}$	Total number of individuals ${N}_{\mathrm{total}}={N}_{\mathrm{group}}\times {G}_{\mathrm{size}}$
	${H}_{\mathrm{seed},z}$, ${H}_{\mathrm{cont},z}$	Proportion of homozygotes (i.e.* AA* or *BB*) in the seeders and contacts, respectively, for group $z$
	$\langle {H}_{\mathrm{seed}}\rangle$, $\langle {H}_{\mathrm{cont}}\rangle$	Average proportion of homozygotes across groups
	${\chi }_{\mathrm{seed},z}$, ${\chi }_{\mathrm{cont},z}$	Homozygote balance (i.e. the proportion of *AA* individuals minus the proportion of *BB* individuals) in the seeders and contacts, respectively. E.g. ${\chi }_{\mathrm{seed}}$ =1 (− 1) if the seeder population consists of *AA* (*BB*) individuals only, and ${\chi }_{\mathrm{seed}}$ = 0 if the seeder population consists of an equal number of *AA* and *BB* individuals
	$\langle {\chi }_{\mathrm{seed}}\rangle$, $\langle {\chi }_{\mathrm{cont}}\rangle$	Average homozygote balance across groups
Population-wide epidemiological parameters	$\beta$	Population average transmission rate
	$\gamma$	Population average recovery rate
	$k$	Shape parameter that characterises the dispersion in infection durations of different individuals
Individual-based epidemiological traits for individual $j$	${\lambda }_{j}$	Force of infection (probability per unit time to become infected)
	${w}_{j}$	Mean of gamma distributed recovery time
	${g}_{j}$, ${f}_{j}$,${r}_{j}$	Fractional deviation in susceptibility, infectivity and recoverability
SNP	${g}_{j}^{\mathrm{SNP}}$, ${f}_{j}^{\mathrm{SNP}}$,${r}_{j}^{\mathrm{SNP}}$	SNP-based contribution to ${g}_{j}$, ${f}_{j}$,${r}_{j}$
	${a}_{g}$, ${a}_{f}$,${a}_{r}$	SNP effects, i.e. half the change in ${g}_{j}$, ${f}_{j}$, ${r}_{j}$ comparing the *AA* and *BB* genotypes
	${\Delta }_{g}$, ${\Delta }_{f}$,${\Delta }_{r}$	Scaled dominance factors (1 = *A* is completely dominant over *B, -1* = *B* is dominant over A, 0 = no dominance)
Fixed effects	${\mathbf{b}}_{{\varvec{g}}}$, ${\mathbf{b}}_{{\varvec{f}}}$,${\mathbf{b}}_{{\varvec{r}}}$	Vectors of fixed effects for the three traits
	$\mathbf{X}$	Design matrix for fixed effects
Residuals	${{\varvec{\upvarepsilon}}}_{{\varvec{g}}}$, ${{\varvec{\upvarepsilon}}}_{{\varvec{f}}}$,${{\varvec{\upvarepsilon}}}_{{\varvec{r}}}$	Residual contributions to $\mathbf{g}$, $\mathbf{f}$, $\mathbf{r}$ (coming from sources other than the SNP, fixed or group effects)
	${\varvec{\Sigma}}$	Covariance matrix of residual contributions
Group effects	${G}_{z}$	Group effects (accounts for differences in transmission rates in different contact groups)
	${\upsigma }_{G}$	Standard deviation in group effects
Bayesian model	$\uptheta$	Set of all model parameters
	$\upxi$	Set of all events (infection and recovery / death times) which may be unknown, i.e. latent variables in the model
Other parameters used in the analyses	${N}_{I}$	Total number of infections during experiment
	$\phi$	Fraction of contacts that become infected
	$h$	Proportion of the total number of infections accounted for by seeders, i.e.$h={N}_{\mathrm{seed}}/({N}_{\mathrm{seed}}+\phi {N}_{\mathrm{cont}})$
	$\mathbf{M}$	Fisher information matrix
	$\langle \dots \rangle$	Average over contact groups
	$\overline{\dots }$	Average over entire infected population (included seeders as well as those individuals infected during epidemics)

Previous studies have shown that the effects of SNPs and other genetic effects for the epidemiological parameters can be inferred from a wide range of available data that can be routinely collected from disease transmission experiments [15, 25, 30, 31]. These may consist of the times at which individuals become infected and/or recover/die, or of results from disease diagnostics tests that provide information on the disease status of individuals at particular points in time. Note that estimates can be inferred even for censored data and it is not required that transmission routes (i.e*.* who infects who) are known [15].

Based on these concepts, for a given disease and epidemiological data from a fixed number of genotyped animals, the optimal experimental design is determined by finding how the numbers of seeders (${N}_{\mathrm{seed}}$), contacts (${N}_{\mathrm{cont}})$, the proportion of homozygotes ${(H}_{\mathrm{seed}}$ and ${H}_{\mathrm{cont}}$), and the homozygote balance ${(\chi }_{\mathrm{seed}}$ and ${\chi }_{\mathrm{cont}})$ should be chosen for each contact group in order to maximise the precision with which SNP effects on susceptibility, infectivity, and recoverability can be estimated. In particular, we identify designs for basic “blocks”, where each “block” consists of one or a number of contact groups with each group having ${H}_{\mathrm{seed}}$, ${H}_{\mathrm{cont}}$, ${\chi }_{\mathrm{seed}}$ and ${\chi }_{\mathrm{cont}}$ specified in an optimal way. These blocks can be replicated one or several times to make up the total contact group number ${N}_{\mathrm{group}}$.

### The genetic-epidemiological model

The infection dynamics within each contact group described above (and illustrated in Fig. 1) can be represented by an epidemiological SIR model, with individuals that are classified as being either susceptible to infection (S), infected and infectious (I), or recovered/removed/dead (R) [14]. The incorporation of individual-based trait variation into this model is taken from [15], which we briefly reiterate here for completeness. The force of infection ${\lambda }_{j}$ (i.e. the probability per unit time that individual $j$ becomes infected) and the mean infection duration ${w}_{j}$ are given by:

$${\lambda }_{j}=\beta {e}^{{G}_{z}}{e}^{{g}_{j}}\sum_{i}{e}^{{f}_{i}},$$

1 $${w}_{j}={(\gamma {e}^{{r}_{j}})}^{-1},$$

where $\beta$ is a transmission rate parameter, $\gamma$ is the population average recovery rate, ${g}_{j}$ and ${r}_{j}$ represent fractional deviations^6^ in the susceptibility and recoverability, respectively, of individual $j$, and ${f}_{i}$ represents the fractional deviation in infectivity of individual $i$ (the sum goes over all currently infected individuals within the same contact group as $j$). Finally, ${G}_{z}$ is a random effect for group $z$, with mean zero and standard deviation ${\upsigma }_{z}$, which accounts for group-specific factors that influence the overall speed of an epidemic in one contact group relative to another (e.g. animals kept in different management conditions or environmental differences). The time for individual $j$ to recover after being infected is taken to be gamma distributed, with mean ${w}_{j}$ and shape parameter $k$ [15]. The individual-based fractional deviations in susceptibility, infectivity, and recoverability are parameterised as follows:

$$\mathbf{g}={\mathbf{g}}^{\mathrm{SNP}}+\mathbf{X}{\mathbf{b}}_{\mathbf{g}}+{{\varvec{\upvarepsilon}}}_{\mathbf{g}},$$

$$\mathbf{f}={\mathbf{f}}^{\mathrm{SNP}}+\mathbf{X}{\mathbf{b}}_{\mathbf{f}}+{{\varvec{\upvarepsilon}}}_{\mathbf{f}},$$

2 $$\mathbf{r}={\mathbf{r}}^{\mathrm{SNP}}+\mathbf{X}{\mathbf{b}}_{\mathbf{r}}+{{\varvec{\upvarepsilon}}}_{\mathbf{r}},$$

where $\mathbf{g}$, $\mathbf{f}$, and $\mathbf{r}$ are vectors (with an element for each individual) that are decomposed into ${\mathbf{g}}^{\mathrm{SNP}}$, ${\mathbf{f}}^{\mathrm{SNP}}$, and ${\mathbf{r}}^{\mathrm{SNP}}$, which include the effects from the SNP under investigation, and fixed effects ${\mathbf{b}}_{\mathbf{g}}$, ${\mathbf{b}}_{\mathbf{f}}$, and ${\mathbf{b}}_{\mathbf{r}}$, where $\mathbf{X}$ is a design matrix (e.g. to account for sex differences in the traits or vaccination status).

The residuals ${\varvec{\upvarepsilon}}={({\varvec{\upvarepsilon}}}_{\mathbf{g}},\boldsymbol{ }{{\varvec{\upvarepsilon}}}_{\mathbf{f}}, {{\varvec{\upvarepsilon}}}_{\mathbf{r}})$ in Eq. (2) account for contributions from all SNPs, excluding the one being investigated, from other sources of polygenic variation, individual permanent non-genetic effects, and environmental effects. These residuals are taken to be multivariate-normal distributed with zero mean and covariance matrix $\mathbf{I}\otimes{\varvec{\Sigma}}$, where $\mathbf{I}$ is the identity matrix, reflecting no correlation between individuals, and ${\varvec{\Sigma}}$ is a 3 × 3 covariance matrix that characterises potential correlations between the three epidemiological traits.^7^ The residual structure does not explicitly distinguish between random genetic and environmental effects, and relies on the assumption that individuals are distributed randomly with regards to the genetic effects on the epidemiological traits that are not captured by the SNP under consideration (see “Discussion” for relaxing this assumption).

The SNP contribution to the traits for individual $j$ is dependent on $j$’s genotype in the following way:

3 $$\left.\begin{array}{ccc}\qquad{a}_{g}& \qquad{ a}_{f}& \qquad{ a}_{r}\\ {g}_{j}^{\mathrm{SNP}}={a}_{g}{\Delta }_{g}& {f}_{j}^{\mathrm{SNP}}={a}_{f}{\Delta }_{f}& {r}_{j}^{\mathrm{SNP}}= {a}_{r}{\Delta }_{r}\\ \qquad{ -a}_{g}& \qquad{ -a}_{f}& \qquad{ -a}_{r}\end{array}\right\} \begin{array}{c}\mathrm{if}\, j\,\mathrm{is}\, AA\\ \mathrm{if}\,j\,\mathrm{is} AB\\ \mathrm{if} j\,\mathrm{is}\,BB\end{array},$$

where ${a}_{g}$, ${a}_{f}$, and ${a}_{r}$ are half the difference in trait values between the *AA* and *BB* homozygote genotypes and ${\Delta }_{g}$, ${\Delta }_{f}$ and ${\Delta }_{r}$ represent the degree of dominance (a value of 1 (− 1) corresponds to complete dominance of the *A* (*B*) allele over the *B* (*A*) allele, whereas absence of dominance is represented by a value of 0) [33].

The model in Eqs. (1–3) contains numerous parameters, but from the point of view of establishing SNP-based associations, the key quantities are ${a}_{g}$, ${a}_{f}$, and ${a}_{r}$, which are subsequently referred to as the “SNP effects” and characterise the changes in susceptibility, infectivity, and recoverability associated with different SNP genotypes (note, ${\Delta }_{g}$, ${\Delta }_{f}$ and ${\Delta }_{r}$ are also important if dominance is of particular interest, as discussed later).

## Results

Given data from a disease transmission experiment, inference can be used to estimate model parameters. Assuming an uninformative flat prior, posterior estimates and associated uncertainties can be captured by a multivariate probability distribution called the likelihood. The precision for each parameter is characterised by the posterior standard deviation (SD) in the corresponding marginalised likelihood.^8^ We begin by deriving analytical expressions for the SD of SNP effects for the three epidemiological traits under some simplifying assumptions (the infection and recovery times of infected individuals are known, effect sizes are relatively small, and fixed effects, group effects, and residuals are all ignored) which provides first insights for how precisions are affected by the experimental design. We then investigate how to maximise these precisions by optimizing this design.

For validation, analytically derived SDs were compared against inferred values from simulated epidemic and genetic data for different experimental designs (for details on the simulation methodology and protocols used for the generation of graphs see Additional file 1). Inference was performed using the software SIRE [15], which incorporates a Bayesian methodology that is flexible to different data types, can account for uncertainty in a statistically consistent way, and has been found to produce unbiased estimates for model parameters. Behaviour when the various assumptions outlined above are violated is also investigated (see Table 2 for a summary of all model and data scenarios considered).

**Table 2:** Data/model scenarios

Data	Design	Residual	Group effect	Fixed effect	Information source
Inf. + Rec	Single group (no dominance estimate)	✕	✕	✕	Figure 3
Inf. + Rec	Pure (no dominance estimate)	✕	✕	✕	Figure 4
Inf. + Rec	Mixed (no dominance estimate)	✕	✕	✕	Figure 5
Inf. + Rec	Pure/mixed (no dominance estimate)	✓	✓	✓	Figure 6
Inf. + Rec	Pure (dominance estimate)	✕	✕	✕	Additional file 9: Fig. S3
Inf. + Rec	Mixed (dominance estimate)	✕	✕	✕	Additional file 10: Fig. S4
Inf. + Rec	Pure/mixed (no dominance estimate)	✓	✕	✕	Additional file 11: Figs. S5–S8
Inf. + Rec	Pure/mixed (no dominance estimate)	✕	✓	✕	Additional file 11: Figs. S5–S7
Inf. + Rec	Pure/mixed (no dominance estimate)	✕	✕	✓	Additional file 11: Figs. S5–S7
Rec. (no Inf.)	Pure/mixed (no dominance estimate)	✕	✕	✕	Additional file 11: Figs. S5–S7
Periodic DS checks	Pure/mixed (no dominance estimate)	✕	✕	✕	Additional file 11: Figs. S5–S7
Inf. + Rec	Pure/mixed (no dominance estimate)	✓	✓	✓	Additional file 11: Figs. S5–S7
Inf. + Rec	Pure/mixed (dominance estimate)	✓	✓	✓	Additional file 12: Fig. S9
Inf. + Rec	HWE	✕	✕	✕	Additional file 15: Fig. S12

**Fig. 2:**
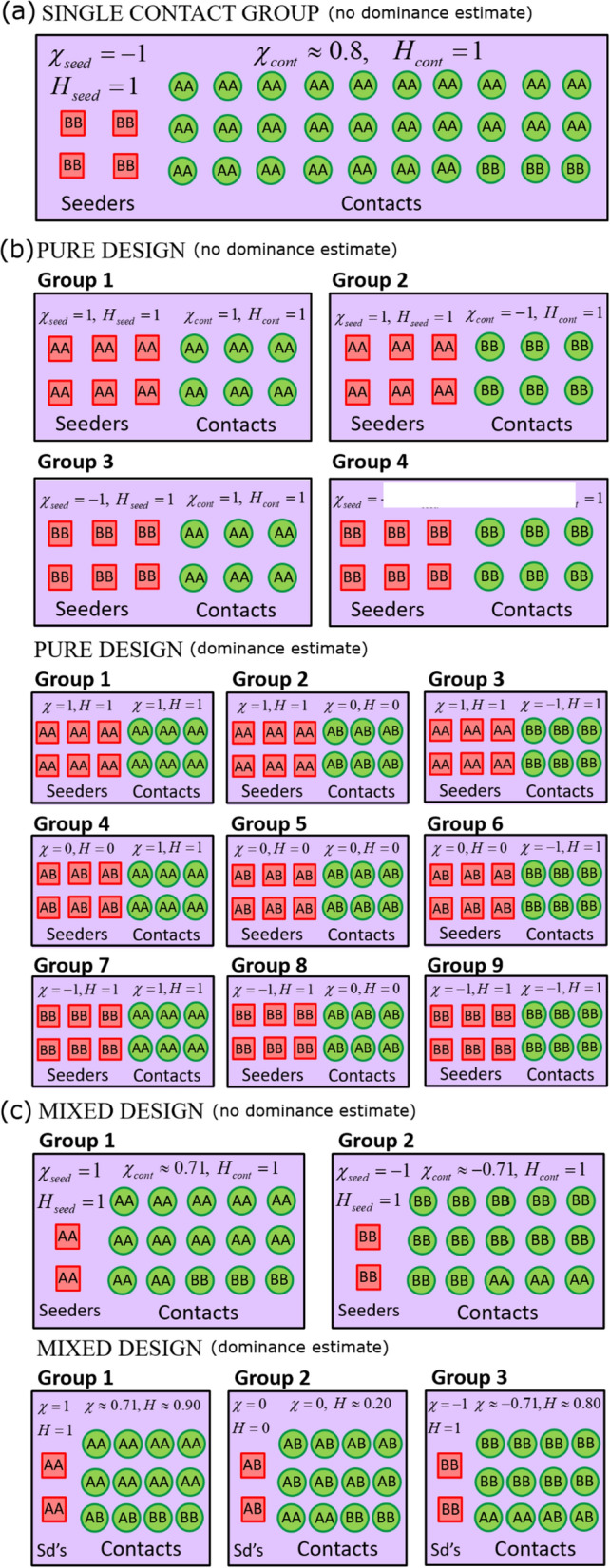
Optimal experimental designs. This figure shows the optimal composition of the seeder and contact populations for different experimental designs: **a** Single contact group design: ~ 15% of individuals are seeders, where seeders have genotype *BB* (or *AA*) and contacts predominately have genotype *AA* (or *BB*), with ~ 10% *BB*, to allow for estimation of the susceptibility SNP effect ${a}_{g}$. Estimation of dominance was found to be challenging using only a single contact group (not shown). **b** Multiple groups “pure” design: ~ 47% of individuals are seeders. Seeders and contacts consist of different combinations of *AA* and *BB* across groups (and *AB* when dominance is investigated). **c** Multiple groups “mixed” design: a small number of individuals are seeders (typically two or three, sufficient to initiate epidemics). When dominance is not investigated, there is a 83%/17% split in *AA*/*BB* individuals in the contact population in group 1 and vice-versa in group 2. When dominance is investigated, there is a 80%/10%/10% split in *AA*/*AB*/*BB* individuals in the contact population in group 1, and these proportions are permuted to define the two other groups. Optimisation of these designs was (for the most part) based on maximising the precision with which the infectivity SNP effect ${a}_{f}$ can be estimated (since this was generally the most difficult trait to estimate). However in cases where maximal precision for ${a}_{f}$ corresponds to minimal precision for ${a}_{g}$, values are chosen to give equal precision to the two (e.g. ~ 10% *BB* in **a**, as discussed in the paper). The percentages above are, to a large extent, independent of ${R}_{0}$ (see Additional file 8) or other factors in the model/data (see Additional file 11). For reference the optimal homozygote balance ${\chi }_{\mathrm{seed}}$ and ${\chi }_{\mathrm{cont}}$ (i.e. proportion of *AA minus BB* individuals) and homozygosity ${H}_{\mathrm{seed}}$ and ${H}_{\mathrm{cont}}$ (i.e. proportion of *AA plus BB* individuals) are shown for each design (the ‘$\approx$’ symbols indicate that these are optimal values to be aimed for, accounting for the fact that the number of individuals is discrete). The same basic designs can be replicated multiple times within an experiment. Note that the results equally apply to the estimation of non-genetic factors, e.g. vaccination effects (*AA* replaced with “Vac.” and *BB* replaced with “Unvac.” and dominance not applicable). The spatial separation between seeders and contacts in this diagram is for illustrative purposes only

This table summarises all the data/model scenarios used in this paper. The columns are as follows: *Data* (“Inf. + Rec.” means that infection and recovery times of all individuals are assumed to be known exactly, “Rec.” means only recovery times are known, and “Periodic DS checks” means the disease status of individuals is periodically checked); *Design* (this includes the five optimal designed illustrated in Fig. 2 as well as “HWE”, in which individuals are randomly allocated genotypes assuming Hardy–Weinberg equilibrium); *Residual* (a tick (✓) is indicated if the model incorporates the residuals ${\varvec{\upvarepsilon}}$ =(${{\varvec{\upvarepsilon}}}_{{\varvec{g}}}$, ${{\varvec{\upvarepsilon}}}_{{\varvec{f}}}$, ${{\varvec{\upvarepsilon}}}_{{\varvec{r}}}$) in Eq. (2)); *Group effect* (a tick (✓) is indicated if the model incorporates the random group effect ${G}_{z}$ in Eq. (1)); *Fixed effect* (a tick (✓) is indicated if the model incorporates a fixed effect $\mathbf{b}$ = (${\mathbf{b}}_{{\varvec{g}}}$, ${\mathbf{b}}_{{\varvec{f}}}$, ${\mathbf{b}}_{{\varvec{r}}}$) in Eq. (2)); and *Information source* (indicates the figure in the main text and in Additional files that relates to the corresponding scenario)

### Analytical expressions

#### SNP effects for susceptibility and infectivity

Based on the model presented in the previous section and known infection and recovery times, it is possible to analytically approximate the marginalised likelihood for the two variables ${a}_{g}$ and ${a}_{f}$ as a two dimensional multivariate-normal distribution with the inverse covariance matrix given by the following 2 × 2 Fisher information matrix (see Additional file 2 for a derivation of this expression):

4 $$\mathbf{M}={N}_{\mathrm{group}}\phi {N}_{\mathrm{cont}}\left[\begin{array}{cc}\langle {H}_{\mathrm{cont}}\rangle -{\langle {\chi }_{\mathrm{cont}}\rangle }^{2}& \mathrm{Var}({\chi }_{\mathrm{cont}})\\ \mathrm{Var}({\chi }_{\mathrm{cont}})& \mathrm{Var}({\chi }_{\mathrm{cont}})\end{array}\right]+{N}_{\mathrm{group}}{N}_{\mathrm{seed}}\left[\begin{array}{cc}0& W\\ W& (2W+Y)\end{array}\right],$$

where

$$W=-\mathrm{log}\left(h\right)\langle \left({\chi }_{\mathrm{cont}}-\langle {\chi }_{\mathrm{cont}}\rangle \right)\left({\chi }_{\mathrm{seed}}-{\chi }_{\mathrm{cont}}\right)\rangle ,$$

5 $$Y=\left(1-h\right)\langle {\left({\chi }_{\mathrm{seed}}-{\chi }_{\mathrm{cont}}\right)}^{2}\rangle -\frac{{N}_{\mathrm{seed}}}{{\phi N}_{\mathrm{cont}}}{\mathrm{log}}^{2}\left(h\right){\langle {\chi }_{\mathrm{seed}}-{\chi }_{\mathrm{cont}}\rangle }^{2}.$$

The parameters are defined as follows: ${N}_{\mathrm{group}}$ is the number of groups, $\phi$ represents the fraction of contacts that ultimately become infected,^9^$h={N}_{\mathrm{seed}}/({N}_{\mathrm{seed}}+{\phi N}_{\mathrm{cont}})$ is the proportion of infected individuals that are seeders at the end of the experiment, ${H}_{\mathrm{cont}}$ gives the proportion of homozygous contacts (i.e. proportion of *AA* plus BB), and ${\chi }_{\mathrm{seed}}$ and ${\chi }_{\mathrm{cont}}$ give the homozygote balance (i.e. proportion of *AA* minus *BB*) in the seeders and contacts, respectively. Note that ${H}_{\mathrm{cont}}$, ${\chi }_{\mathrm{seed}}$, and ${\chi }_{\mathrm{cont}}$ have (potentially) different values for each group. The angle brackets in Eqs. (4) and (5) denote averaging of these quantities across groups, and $\mathrm{Var}({\chi }_{\mathrm{cont}})$ gives the variance of the homozygote balance for the contacts between groups.

An important point to take from Eq. (4) is that $\mathbf{M}$ is actually the sum of two matrices. The first corresponds to information provided by infections that occur during the course of the observed epidemics (note this contribution contains a factor ${N}_{\mathrm{group}}\phi {N}_{\mathrm{cont}}$, which is the total expected number of infected contacts) and the second comes from information gained from the pattern of infections early on in the epidemics (since we know these are mainly caused by seeders) as a result of differences in genetic makeup between seeders and contacts in the initial conditions^10^ (note this contains a factor giving the total number of initially infected individuals ${N}_{\mathrm{group}}{N}_{\mathrm{seed}}$).

Inversion of the Fisher information matrix defined in Eq. (4) leads to an estimate for the posterior covariance matrix (see Additional file 3 for further details). The square root of the diagonals of this matrix provide posterior SDs for the parameters ${a}_{g}$ and ${a}_{f}$:

6 $$\mathrm{SD\, in\,}{a}_{g}\cong \frac{1}{\sqrt{{N}_{\mathrm{group}}\phi {N}_{\mathrm{cont}}\left(\langle {H}_{\mathrm{cont}}\rangle -{\langle {\chi }_{\mathrm{cont}}\rangle }^{2}\right)-{N}_{\mathrm{group}}\frac{{\left(\phi {N}_{\mathrm{cont}}\mathrm{Var}\left({\chi }_{\mathrm{cont}}\right)+{N}_{\mathrm{seed}}W\right)}^{2}}{\phi {N}_{\mathrm{cont}}\mathrm{Var}\left({\chi }_{\mathrm{cont}}\right)+{N}_{\mathrm{seed}\left(2\mathrm{W}+\mathrm{Y}\right)}}}},$$

7 $${\mathrm{SD\,in\,}a}_{f}\cong \frac{1}{\sqrt{{N}_{\mathrm{group}}{N}_{\mathrm{seed}}\left(Y+\frac{2\left( \langle {H}_{\mathrm{cont}}\rangle -\langle {\chi }_{\mathrm{cont}}^{2}\rangle \right)W-\frac{{N}_{\mathrm{seed}}}{\phi {N}_{\mathrm{cont}}}{W}^{2}}{ \langle {H}_{\mathrm{cont}}\rangle - {\langle {\chi }_{\mathrm{cont}}\rangle }^{2}} \right)-{N}_{\mathrm{group}}\phi {N}_{\mathrm{cont}}\frac{\langle {H}_{\mathrm{cont}}\rangle -\langle {\chi }_{\mathrm{cont}}^{2}\rangle }{\langle {H}_{\mathrm{cont}}\rangle - {\langle {\chi }_{\mathrm{cont}}\rangle }^{2}}\mathrm{Var}\left({\chi }_{\mathrm{cont}}\right)}}$$

These rather unwieldy expressions reflect a complex confounding between estimating susceptibility and infectivity SNP effects. They show that precisions of parameter estimates depend not only on the number of seeders and contacts, but also on the genetic composition of each group for the SNP.

In spite of their apparent complexity, a number of important design lessons can be drawn from Eqs. (6) and (7): (1) they both scale as ${N}_{\mathrm{group}}^{-1/2}$ (which means that increasing the number of groups by a factor of four halves the SDs)^11^; (2) a higher proportion of infections $\phi$ implies greater precision (the more contacts that become infected, the greater the available information on which inferences can be based, although uninfected individuals do provide some information about their susceptibility); (3) for large ${N}_{\mathrm{cont}}$ and fixed ${N}_{\mathrm{seed}}$, we observe that both SDs scale as ${N}_{\mathrm{cont}}^{-1/2}$, meaning that greater precision results from a larger contact population (a notable exception to this is the case of a single contact group, for which the variance $\mathrm{Var}({\chi }_{\mathrm{cont}})$ in Eq. (7) becomes exactly zero, and so this term vanishes); (4) the SDs do not depend on the effect sizes (i.e. ${a}_{g}$ and ${a}_{f}$ do not appear in Eqs. (6) and (7)); and (5) precision is maximised when the homozygosity in the contacts is 1, i.e. $\langle {H}_{\mathrm{cont}}\rangle =1$, referring to experiments that only contains *AA* and *BB* contact individuals (as *AB* heterozygotes provide less information because they dilute the relative effects of the *A* and *B* alleles in the case of zero dominance).

#### SNP effects for recoverability

Equivalent analytical expressions for recoverability can be derived (see Additional file 4 for further details). Assuming no dominance, this leads to:

8 $${\mathrm{SD\,in\,}a}_{r}=\frac{1}{\sqrt{k{N}_{\mathrm{group}}({N}_{\mathrm{seed}}+\phi {N}_{\mathrm{cont}})(\overline{H }-{\overline{\chi }}^{2})}},$$

where

9 $$\overline{H }=\frac{{N}_{\mathrm{seed}}\langle {H}_{\mathrm{seed}}\rangle +\phi {N}_{\mathrm{cont}}\langle {H}_{\mathrm{cont}}\rangle )}{{N}_{\mathrm{seed}}+\phi {N}_{\mathrm{cont}}}\, {\rm and}\,\overline{\chi }=\frac{{N}_{\mathrm{seed}}\langle {\chi }_{\mathrm{seed}}\rangle +\phi {N}_{\mathrm{cont}}\langle {\chi }_{\mathrm{cont}}\rangle }{{N}_{\mathrm{seed}}+\phi {N}_{\mathrm{cont}}}$$

represent, respectively, the average homozygosity and homozygote balance for the entire infected population (i.e. including the seeders and the contacts that become infected during the experiment). Note that inclusion of the shape parameter $k$ in Eq. (8) incorporates the fact that recovery dynamics are governed by a peaked gamma distribution.

#### Dominance

So far, we made the assumption of no dominance between *A* and *B* alleles. However, it is worth noting that the analytical results obtained also apply for the case of complete dominance by means of a simple change in parameter definitions. When allele *A* has complete dominance over *B*, the genotypes *AA* and *AB* become indistinguishable, and so the homozygote balance parameters ${\chi }_{\mathrm{seed}}$ and ${\chi }_{\mathrm{cont}}$ can be redefined as the proportion of *AA* and *AB* individuals minus the proportion of *BB* individuals in the seeders and contacts, respectively, and the homozygosity ${H}_{\mathrm{cont}}$ becomes 1.

In the general case, expressions for the SDs in the posterior distributions for ${\Delta }_{g}$, ${\Delta }_{f}$ and ${\Delta }_{r}$ are as follows (see Additional file 5 for details):

10 $${\mathrm{SD\,in\,}}{\Delta }_{g}\cong \frac{1}{\left|{a}_{g}\right|\sqrt{{N}_{\mathrm{group}}\phi {N}_{\mathrm{cont}}\left(\langle {H}_{\mathrm{cont}}\rangle -{\langle {H}_{\mathrm{cont}}\rangle }^{2}\right)}},$$

where $\langle {H}_{\mathrm{cont}}\rangle$ is the average homozygosity of all contact individuals. Interestingly, this expression is optimised when $\langle {H}_{\mathrm{cont}}\rangle =1/2$, irrespective of exactly how the homozygous individuals are distributed across groups. Note that the expression in Eq. (10) diverges to infinity in the limit of no homozygosity ($\langle {H}_{\mathrm{cont}}\rangle =0$) or complete homozygosity ($\langle {H}_{\mathrm{cont}}\rangle =1$), as expected.

For infectivity:

11 $${\rm SD\,in} {\Delta }_{f}\cong \frac{1}{\left|{a}_{f}\right|\sqrt{{N}_{\mathrm{group}}\phi {N}_{\mathrm{cont}}\mathrm{Var}\left({H}_{\mathrm{cont}}\right)+{N}_{\mathrm{group}}{N}_{\mathrm{seed}}\left(2{W}_{H}+{Y}_{H}\right)}},$$

where,

$${W}_{H}=-\mathrm{log}\left(h\right)\langle \left({H}_{\mathrm{cont}}-\langle {H}_{\mathrm{cont}}\rangle \right)\left({H}_{\mathrm{seed}}-{H}_{\mathrm{cont}}\right)\rangle ,$$

12 $${Y}_{H}=\left(1-h\right)\langle {\left({H}_{\mathrm{seed}}-{H}_{\mathrm{cont}}\right)}^{2}\rangle -\frac{{N}_{\mathrm{seed}}}{\phi {N}_{\mathrm{cont}}}{\mathrm{log}}^{2}\left(h\right){\langle {H}_{\mathrm{seed}}-{H}_{\mathrm{cont}}\rangle }^{2},$$

and for recoverability:

13 $${\mathrm{SD\,in}} \Delta_{r}\cong \frac{1}{\left|{a}_{r}\right|\sqrt{k{N}_{\mathrm{group}}\left({N}_{\mathrm{seed}}+\phi {N}_{\mathrm{cont}}\right)\left(\overline{H }-{\overline{H} }^{2}\right)}},$$

where $\overline{H }$ is the average homozygosity over the entire infected population (regardless of their distribution across groups), as defined in Eq. (9).

### Experimental designs

From the outset, it should be emphasised that there is no single optimal experimental design because (1) the optimal design depends on a trade-off in precision between different parameter estimates (a given design that estimates one parameter as precisely as possible may be less precise for other parameters in the model); and (2) practical considerations often restrict what can be implemented (e.g. physical or budget constraints may restrict the number of groups or group sizes^12^).

As will be demonstrated later, the infectivity parameter ${a}_{f}$ is the most difficult of the SNP effects to estimate. It is natural, therefore, to focus on experimental designs that reduce the posterior SD in this parameter as much as possible.^13^ In the case of a single contact group, mathematical minimisation of Eq. (7) can explicitly be performed, leading to a unique optimal solution (see below). However, in the case of multiple groups (with the same overall number of individuals), such minimisation is challenging due to the complexity of the expression. Nevertheless, it was found that individually maximising each of the two terms in the denominator in Eq. (7) led to two contrasting approaches.^14^ As a result, three basic designs for disease transmission experiments emerge, as illustrated in Fig. 2 and discussed in detail below (along with design-specific analytical expressions for the SD in ${a}_{f}$): (1) a design for a single contact group, (2) designs referred to as “pure”, and (3) designs that will be referred to as the “mixed”. For simplicity, in the following we assume that the basic reproduction number ${R}_{0}$ is reasonably high such that most contacts become infected,^15^ i.e. $\phi \approx 1$ (even when this is not the case, the conclusions related to optimal design remain largely unchanged^16^).

Typically to increase experimental power, it is routine to perform multiple replicates of a given experimental design. This possibility is incorporated into the analytical expressions below by virtue of the fact that ${N}_{\mathrm{group}}$ refers to the total number of contact groups across all replicates.

#### Single contact group

**Fig. 3:**
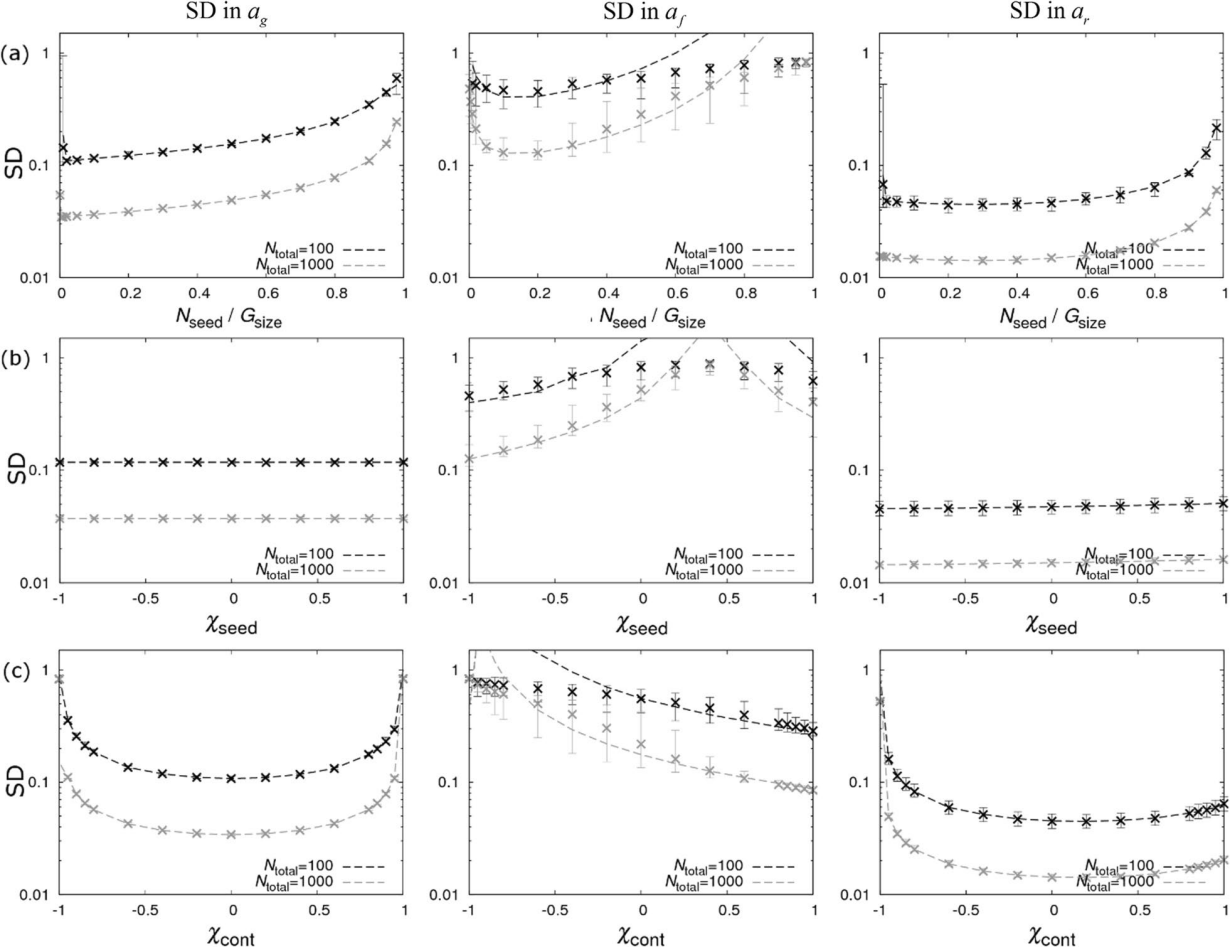
Single contact group design. Precision estimates for the single contact group design (no dominance estimate) in Fig. 2a. The left, middle and right columns show graphs for standard deviations (SDs) in the posterior distributions for SNP effects for susceptibility ${a}_{g}$, infectivity ${a}_{f},$ and recoverability ${a}_{r}$ under different scenarios. **a** The fraction of seeder individuals is varied (arbitrarily fixing ${\chi }_{\mathrm{seed}}=-1$, ${\chi }_{\mathrm{cont}}=0.4$). **b** The composition of SNP genotypes in the seeder population is changed by varying ${\chi }_{\mathrm{seed}}$ (fixing ${N}_{\mathrm{seed}}/{G}_{\mathrm{size}}=0.15$ and ${\chi }_{\mathrm{cont}}=0.4$). Here the left-hand edge of the graph corresponds to the case when all seeders are *BB* and the right edge is when they are all *AA* (points in between represent a mixture of the two). **c** The composition of SNP genotypes in the contact population is changed by varying ${\chi }_{\mathrm{cont}}$ (fixing ${N}_{\mathrm{seed}}/{G}_{\mathrm{size}}=0.15$ and ${\chi }_{\mathrm{seed}}=-1$). Note that a low SD implies high precision. Dashed lines represent analytical results and crosses refer to posterior estimates from simulated data (see Additional file 1). ${N}_{\mathrm{total}}$ refers to the total number of individuals

Here, we consider the case in which the disease transmission experiment consists of just a single contact group, as illustrated in Fig. 2a. We investigate how the proportion of individuals that are seeders (i.e*.* ${N}_{\mathrm{seed}}/{G}_{\mathrm{size}}$), along with the genetic makeup in the seeders and contacts should be chosen to infer the values for the SNP effects as precisely as possible. This is undertaken by varying each of these quantities in turn while keeping the other two fixed. The results are shown in Fig. 3.

Before describing these graphs in detail, some general points can be made (irrespective of the experimental design). First, agreement between the analytical curves (dashed lines) and the simulation-based results (crosses), (for details see Additional file 1) is generally very good. A notable exception is when the analytic expressions predict very large SD (which manifests itself mostly for ${a}_{f}$ because of the large SD associated with this parameter). This discrepancy arises because the assumption of small SNP effects used in the analysis becomes invalid. In the regime in which SNP effect sizes are not small, analytic expressions tend to be conservative in that they suggest that designs are poorer than they actually are. This shortcoming, however, is not very restrictive because it occurs in experimental designs in which very little information is available anyway (which is not how an experimenter would aim to design their experiment; in addition the analytical results would warn against such designs).

Second, the SD of the recoverability estimates, ${a}_{r}$ (right-hand column of the graphs in Fig. 3), are generally lower than those of the susceptibility estimates ${a}_{g}$ (left-hand column), which are themselves lower than the infectivity estimates (middle column). This was already noted in [15] and implies that the SNP-based differences in recoverability are the easiest to identify, followed by those in susceptibility, with SNP-based differences in infectivity the hardest to estimate.

In the case of recoverability, the reason that estimates for ${a}_{r}$ are significantly more precise than for the other two traits is because recovery times are usually less dispersed (they typically follow a peaked gamma distribution) than infection times (which follow a wide exponential distribution). Estimates of ${a}_{r}$ also do not suffer from confounding between ${a}_{g}$ and ${a}_{f}$ which can make them much less certain in many circumstances.

Lastly, since precision is expected to scale as the square root of the total number of individuals, the SDs for an experiment with 1000 individuals are expected to be a factor √10 = 3.2 times smaller than those for an experiment containing 100 individuals. This can be seen in Fig. 3 by an approximately constant distance between the black and grey dashed curves (note the log scale on the *y*-axis).

We now consider optimising the proportions of seeder and contact individuals in the single contact group design for maximum precision. Figure 3a shows the case of varying the proportion of seeder individuals for a given^17^ genetic makeup of the seeder and contact populations. Looking at the results for the SD in ${a}_{g}$ (left-hand graph in Fig. 3a) we see, generally speaking, that the SD reduces for fewer seeders, which is not surprising given that information regarding susceptibility comes from the infection times of the contact individuals. For a very small number of seeders, there is also the possibility of epidemic extinction, which leads to an increase in the SD (see Additional file 6: Fig. S10). Consequently, careful consideration must be given as to how many seeders are necessary to successfully instigate an epidemic within a group (this will depend on ${R}_{0}$).

In contrast, the SD in the SNP effect for infectivity, ${a}_{f}$ (as shown by the middle graph in Fig. 3a), has a different optimum. For a single contact group, the analytical expression in Eq. (7) simplifies to:

14 $${\mathrm{SD\,in\,}}{a}_{f}\cong \frac{1}{\sqrt{{N}_{\mathrm{total}}\left(h\left(1-h\right)-\frac{{h}^{2}}{1-h}{\mathrm{log}}^{2}\left(h\right)\right){\left({\chi }_{\mathrm{seed}}-{\chi }_{\mathrm{cont}}\right)}^{2}}},$$

where, due to the approximation $\phi \approx 1$, $h={N}_{\mathrm{seed}}/{G}_{\mathrm{size}}$ is the proportion of seeder individuals in the group (corresponding expressions for the other two traits are in Additional file 7). The functional dependence on $h$ gives the profile in the graph, which reaches its minimum when $h=0.15$ (hence the choice of 15% of seeders mentioned in Fig. 2a). In fact, numerical analysis shows that, to a good approximation, this result remains true irrespective of the value of ${R}_{0}$ (on which $\phi$ depends), as demonstrated in Additional file 8: Fig. S2.

Figure 3b shows how the SDs changes with the genetic makeup of the seeder population, as characterised by ${\chi }_{\mathrm{seed}}$ (arbitrarily fixing ${\chi }_{\mathrm{cont}}$ = 0.4 and using the optimum proportion of seeders, $h=0.15$). We find, however, that this genetic makeup has very little effect on the precision of estimates of ${a}_{g}$ and ${a}_{r}$, but a large effect on the SD in ${a}_{f}$. This is because information regarding infectivity actually *relies* on differences in the genetic makeup (i.e. the proportions of *AA*, *BB,* and *AB* individuals) between the seeders and contacts,^18^ which results in variation in the genetic composition of the group of infected individuals over time. This is driven by the term ${({\chi }_{\mathrm{seed}}-{\chi }_{\mathrm{cont}})}^{2}$ in Eq. (14), with the analytical curves diverging in the limit ${\chi }_{\mathrm{seed}}\to {\chi }_{\mathrm{cont}}$.

The reason that differences in the genetic makeup between the seeders and contacts provide information about the relative infectivity of *A* and *B* alleles can be explained intuitively as follows. Suppose ${\chi }_{\mathrm{seed}}=-1$, such that the seeders are only *BB* individuals and ${\chi }_{\mathrm{cont}}=1$, such that the contacts are only *AA* individuals. Because susceptible individuals become infected as the epidemic progresses, the infected population becomes more and more a mixture of *AA* and *BB* individuals. Thus, a comparison of how quickly^19^ the epidemic develops early on as compared to later gives direct evidence for the relative infectivity of *AA* compared to *BB* individuals, and hence of the *A* compared to the *B* allele^20^ (so the precision at different ${\chi }_{\mathrm{seed}}$ depends on the value of ${\chi }_{\mathrm{cont}}$, and vice versa).

Figure 3c shows a contrasting design space, in which the genetic makeup of the *contact* population varies between designs; thus we vary ${\chi }_{\mathrm{cont}}$ (fixing ${\chi }_{\mathrm{seed}}=-1$ and $h=0.15$). Here we find that the SD in ${a}_{f}$ is minimised when ${\chi }_{\mathrm{cont}}=1$, because this makes the contact population as genetically different from the seeder population as possible. However, unfortunately here the SD in the susceptibility SNP effect, ${a}_{g}$ diverges because there are no *BB* contacts and so no information regarding their susceptibility. Therefore, to attain a reasonable precision for ${a}_{g}$, ${\chi }_{\mathrm{cont}}$ must be less than 1. A sensible choice is ${\chi }_{\mathrm{cont}}\approx 0.8$, which, as can be seen from the right-hand side of Fig. 3c, leads to only a modest increase in the SD in ${a}_{f}$, with the SD in ${a}_{g}$ smaller than that for ${a}_{f}$. This corresponds to around 10% *BB* individuals in the contact population, as quoted in Fig. 2a.

The single contact group design contains only *AA* and *BB* individuals and, therefore, no information regarding the dominance relationship between the *A* and *B* alleles is provided. Introduction of *AB* individuals into the seeders and contacts can inform ${\Delta }_{g}$ and ${\Delta }_{r}$, but it turns out that almost nothing can be inferred regarding the infectivity dominance factor ${\Delta }_{f}$ (results not shown). Consequently, here this possibility is not investigated further.

### Multiple contact groups: “pure” design

Perhaps the most intuitive experimental design, which we term the “pure” design, is illustrated in Fig. 2b. When dominance is not being investigated, this consists of running replicates that consist of four groups in which the seeders and contacts are each genetically homogeneous (i.e. “pure”) but have different combinations of genotypes *AA* and *BB* across those groups. Such an approach is appealing because it allows conclusions to be easily drawn directly from the data. For example, if epidemics progress significantly faster in groups that contain *AA* seeders (i.e. groups 1 and 2 in Fig. 2b) compared to those that contain *BB* seeders (i.e. groups 3 and 4), this provides direct evidence that allele *A* confers greater infectivity than allele *B* (largely regardless of their relative susceptibility). Similarly, the relative susceptibility of allele *A* compared to *B* can be found by comparing the relative epidemic speeds of groups where the infection was initiated by the same seeder genotypes, but the genotypes of the contact individuals differ (i.e. comparing groups 1,3, and 2,4 in Fig. 2b). This design was implemented in [25] to estimate genotypic effects for a specific resistance marker on susceptibility, infectivity, and recoverability.

**Fig. 4:**
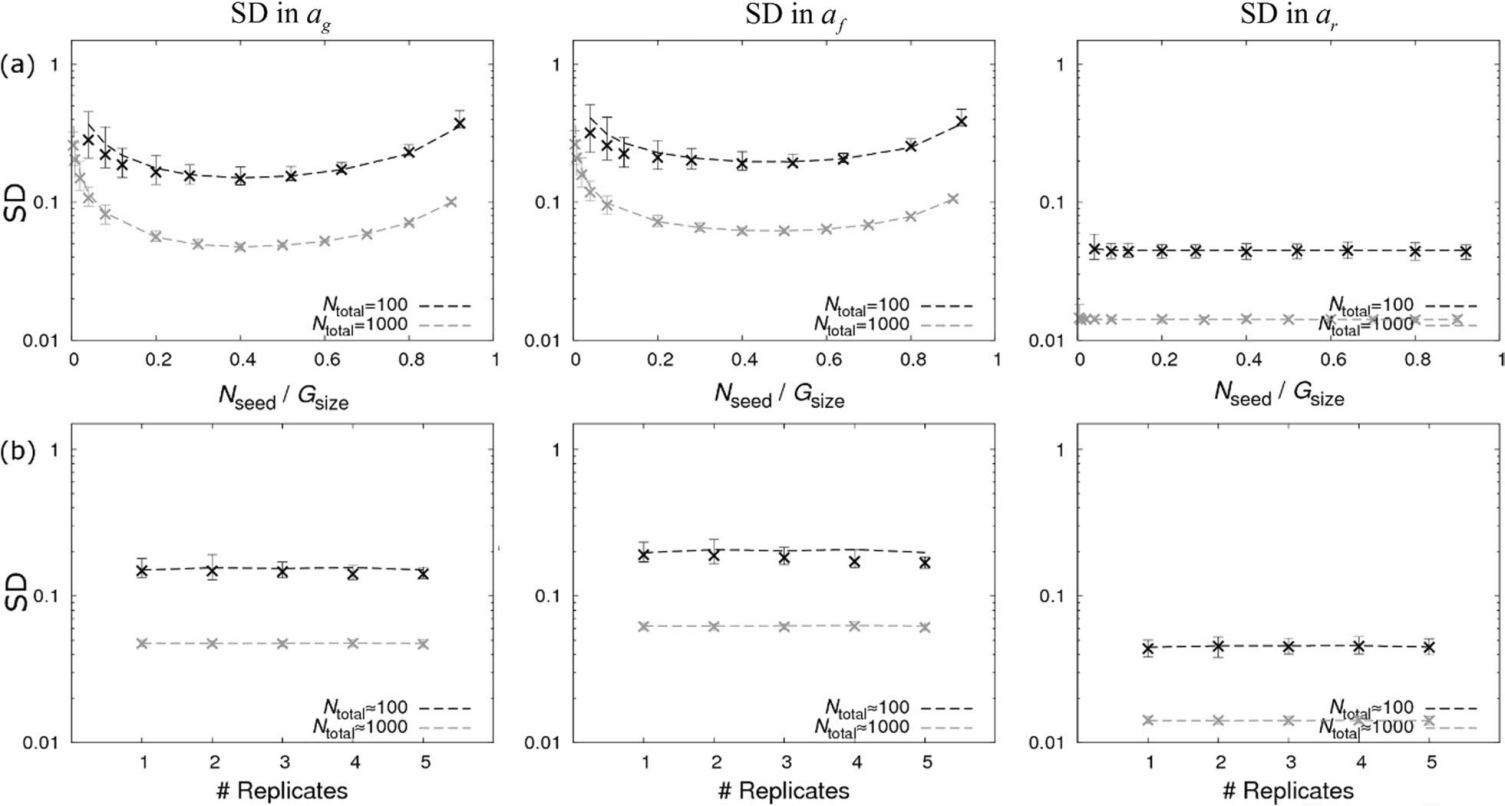
The “pure” design. Precision estimates for the pure design (no dominance estimate) consisting of four contact groups per replicate with homogeneous seeder/contact SNP genotypes illustrated in Fig. 2b. The left, middle and right columns show graphs for standard deviations (SDs) in the posterior distributions for the SNP effects for susceptibility ${a}_{g}$, infectivity ${a}_{f}$ and recoverability ${a}_{r}$ under different scenarios. **a** The fraction of seeder individuals in each contact group is varied. **b** The number of experimental replicates, each consisting of four contact groups, is varied (with smaller groups sizes keeping the total number of individuals approximately fixed). Dashed lines represent analytical results and crosses refer to posterior estimates from simulated data (see Additional file 1). ${N}_{\mathrm{total}}$ refers to the total number of individuals

Figure 4a shows how the SDs in the SNP effects change as the fraction of seeder individuals varies. For the pure design, Eq. (7) simplifies to:

15 $$\mathrm{SD\,of\,}{a}_{f}\cong \frac{1}{\sqrt{{N}_{\mathrm{total}}\left(2h\left(1-h\right)-\frac{{h}^{2}}{1-h}{\mathrm{log}}^{2}\left(h\right)\right)}},$$

which is optimised when $h=0.47$, i.e. 47% of individuals should be seeders (expressions for the other two traits are in Additional file 9). Again, this conclusion largely holds regardless of ${R}_{0}$, as demonstrated in Additional file 8, even when the proportion of contacts that become infected substantially reduces as ${R}_{0}\to 1$. Comparing optimum solutions for the same number of individuals, we find that the SD in the SNP effect for infectivity, ${a}_{f},$ is around 1.6 times smaller for the pure design in Eq. (15) than for the single contact group design in Eq. (14). This means that disease transmission experiments using the pure design require 2.5 times fewer individuals to generate equivalent precision. This highlights the point that multiple groups substantially improve parameter estimates for infectivity.

The design with no dominance estimate in Fig. 2b consists of four groups. Suppose that, instead, we design an experiment with eight groups by copying the same basic design over two replicates (each containing half the number of individuals). Such an approach is investigated in Fig. 4b, where the number of replicates is changed for an (approximately) fixed total number of individuals. We find almost no variation in inference precision, which suggests that the experimenter is free to choose the number of individuals per group (as usually dictated by practical considerations), with the number of replicates driven by the total number of individuals available for the experiment. It should be noted, however, that design replication does play an important role in moderating the potential reduction in precision caused by group effects (as well as other systematic effects) that should be accounted for. This is discussed later in the “Realistic model and data scenarios” section.

The pure design above proved effective at precisely estimating ${a}_{g}$, ${a}_{f}$, and ${a}_{r}$. However, because it does not contain *AB* individuals, it cannot provide information about the dominance relationship between the *A* and *B* alleles. To address this, here we introduce the pure design with dominance estimation, as illustrated by the second design in Fig. 2b. This consists of running replicates of nine groups, in which seeder and contact populations are each genetically homogeneous *or* “pure” within each group (i.e. the individuals within these groupings have the same genotype^21^) but take different seeder/contact combinations of *AA*, *AB,* and *BB* (for further details see Additional file 9). The corresponding analytical equation for the SD in ${a}_{f}$ is given by Eq. (15) multiplied by a constant factor √(3/2) ≈ 1.2. Hence, this design leads to only a modest reduction in precision of SNP effect size estimates, while having the benefit of also providing dominance parameter estimates.

### Multiple contact groups: “mixed” design

The so-called “mixed design” uses replicates of the design illustrated in Fig. 2c. Here, the contacts in group 1 contain a mixture of genotypes and the contacts in group 2 contain the complementary mixture (with *AA* and *BB* interchanged). Unlike the pure design, the mixed design does not rely on a large number of seeders (in fact the smaller the better).

**Fig. 5:**
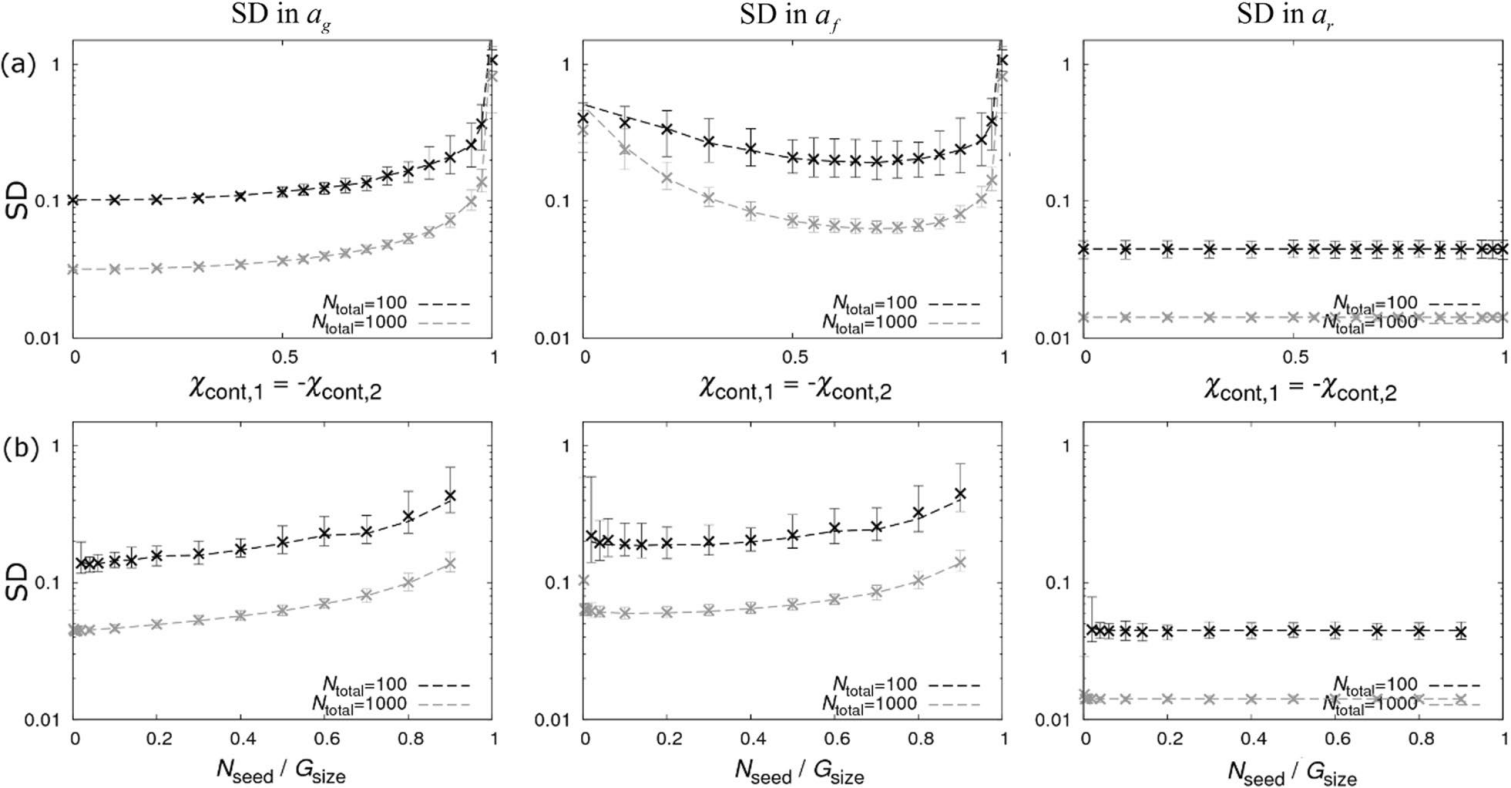
The “mixed” design. Precision estimates for the mixed design (no dominance estimate) consisting of two contact groups per replicate with homogeneous seeder SNP genotypes and heterogeneous contact SNP genotypes illustrated in Fig. 2c. The left, middle and right columns show graphs for standard deviations (SDs) in the posterior distributions for SNP effects for susceptibility ${a}_{g}$, infectivity ${a}_{f},$ and recoverability ${a}_{r}$ under different scenarios. **a** The composition of SNP genotypes in the contact population in group 1 is changed by varying χ_cont,1_ while using the opposite value ${\chi }_{\mathrm{cont},2}=-{\chi }_{\mathrm{cont},1}$ in group 2 and ${N}_{\mathrm{seed}}=3$. **b** The fraction of seeders is varied (fixing ${\chi }_{\mathrm{cont},2}=-{\chi }_{\mathrm{cont},1}=1/\sqrt{2}$)*.* Dashed lines represent analytical results and crosses come from posterior estimates from simulated data (see Additional file 1). ${N}_{\mathrm{total}}$ refers to the total number of individuals.

Results for the mixed design are shown in Fig. 5. The middle graph in Fig. 5a shows how the SD in ${a}_{f}$ varies as the composition of SNP genotypes in the two contact populations is changed. Assuming the first term in the denominator of Eq. (7) is negligible, which is valid in the limit of few seeders, Eq. (7) simplifies to:

16 $${\rm SD\,in\,a}_{f}\cong \frac{1}{\sqrt{{N}_{\mathrm{total}}\left(1-{\chi }_{\mathrm{cont},1}^{2}\right){\chi }_{\mathrm{cont},1}^{2}}},$$

where ${\chi }_{\mathrm{cont},2}=1-{\chi }_{\mathrm{cont},1}$. This is minimised when ${\chi }_{\mathrm{cont},1}=1/\sqrt{2}$ (or ${\chi }_{\mathrm{cont},1}=-1/\sqrt{2}$), corresponding to 15% *BB* and 85% *AA* in the contact population of group 1 and $\approx$ 15% *AA* and $\approx$ 85% *BB* in the contact population of group 2.^22^ Expressions for the SDs in the SNP effects for susceptibility and recoverability are in Additional file 10.

An intuitive explanation of how this experimental design works is as follows. As with most disease transmission experiments, when an infection occurs it is not known from which individual that infection originates. However, a key feature of the mixed design is that there is a *greater probability* that infections are initiated by *AA* individuals in group 1, simply because there are more of them. Likewise, in group 2, *BB* individuals cause most of the infections. Consequently, if the epidemic in group 1 proceeds more quickly than in group 2, it is tempting to conclude that the *A* allele confers greater infectivity than *B*. However, caution is required due to potential confounding between infectivity and susceptibility (as a similar argument could be made to suggest that the *A* allele confers greater *susceptibility*). Fortunately, this confounding is broken because for each group the relative rate at which *AA* and *BB* individuals become infected gives direct evidence for differences in susceptibility (irrespective of infectivity), so allowing precise estimation of both ${a}_{g}$ and ${a}_{f}$. The right-hand graph in Fig. 5a shows that estimation of the SNP effect on recoverability, ${a}_{r}$, remains the most precise of the three traits.

Figure 5b shows that precision is greatest when the fraction of seeders is small (subject to the extinction problem mentioned earlier). This highlights that the mixed design predominantly gains information from infections within groups, whereas the pure design relies heavily of information gained from the seeders (Fig. 4a).

Again, if no *AB* individuals are present in the mixed design, it cannot be used to provide information regarding dominance. Therefore, for completeness, we also include a mixed design with dominance estimation, as illustrated in Fig. 2c (for further details see Additional file 10).

For comparison, the precision of SNP effects from the five optimal designs in Fig. 2 are given in Table 3.

**Table 3:** Parameter precision estimates

Design	SD in ${{\varvec{a}}}_{{\varvec{g}}}$	SD in ${{\varvec{a}}}_{{\varvec{f}}}$	SD in ${{\varvec{a}}}_{{\varvec{r}}}$	SD in ${{\varvec{\Delta}}}_{{\varvec{g}}}$	SD in ${{\varvec{\Delta}}}_{{\varvec{f}}}$	SD in ${{\varvec{\Delta}}}_{{\varvec{r}}}$
Single group (no dominance estimate)	$\frac{1.08}{\sqrt{{N}_{\mathrm{total}}}}$	$\frac{3.09}{\sqrt{{N}_{\mathrm{total}}}}$	$\frac{1}{\sqrt{{kN}_{\mathrm{total}}}}$	∞	∞	∞
Pure design (no dominance estimate)	$\frac{1.52}{\sqrt{{N}_{\mathrm{total}}}}$	$\frac{1.96}{\sqrt{{N}_{\mathrm{total}}}}$	$\frac{1}{\sqrt{{kN}_{\mathrm{total}}}}$	∞	∞	∞
Pure design (dominance estimate)	$\frac{1.86}{\sqrt{{N}_{\mathrm{total}}}}$	$\frac{2.40}{\sqrt{{N}_{\mathrm{total}}}}$	$\frac{1.22}{\sqrt{{kN}_{\mathrm{total}}}}$	$\frac{2.91}{\left|{a}_{g}\right|\sqrt{{N}_{\mathrm{total}}}}$	$\frac{3.76}{\left|{a}_{f}\right|\sqrt{{N}_{\mathrm{total}}}}$	$\frac{2.60}{\left|{a}_{r}\right|\sqrt{{kN}_{\mathrm{total}}}}$
Mixed design (no dominance estimate)	$\frac{1.41}{\sqrt{{N}_{\mathrm{total}}}}$	$\frac{2}{\sqrt{{N}_{\mathrm{total}}}}$	$\frac{1}{\sqrt{{kN}_{\mathrm{total}}}}$	∞	∞	∞
Mixed design (dominance estimate)	$\frac{1.73}{\sqrt{{N}_{\mathrm{total}}}}$	$\frac{2.45}{\sqrt{{N}_{\mathrm{total}}}}$	$\frac{1.22}{\sqrt{{kN}_{\mathrm{total}}}}$	$\frac{2.60}{\left|{a}_{g}\right|\sqrt{{N}_{\mathrm{total}}}}$	$\frac{3.67}{\left|{a}_{f}\right|\sqrt{{N}_{\mathrm{total}}}}$	$\frac{2.60}{\left|{a}_{r}\right|\sqrt{{kN}_{\mathrm{total}}}}$

This table provides analytically derived estimates for parameter precisions (as measured by the posterior standard deviations (SDs) in the SNP effects ${a}_{g}$, ${a}_{f}$, and ${a}_{r}$ and dominance parameters ${\Delta }_{g}$, ${\Delta }_{f}$, and ${\Delta }_{r}$) for the optimum designs outlined in Fig. 2

### Other fixed effects

Note that our focus here is on estimating SNP effects, but it is important to point out that the analytical results and experimental designs outlined above are equally applicable to quantifying differences in susceptibility, infectivity, and recoverability due to other systematic effects. For example, if the influence of vaccination status is being studied, the *AA* and *BB* genotypes can simply be replaced by “vaccinated” and “unvaccinated” classifications (note that in this case there is no clear analogue of the *AB* genotype, so the dominance designs in Fig. 2b, c become redundant).

### Realistic model and data scenarios

Derivation of the analytical results above made use of some key simplifying assumptions, including infection and recovery times of individuals being precisely known and that the epidemiological traits depend only on the SNP itself (i.e. the residuals and fixed and group effects in Eq. (2) were ignored). Here, we assess the impact of relaxing these assumptions and investigate what implications this has on experimental designs. In particular, five sources of additional variation in the model or data were investigated separately: (1) introducing residual variation in traits, i.e. ${\varvec{\upvarepsilon}}$ in Eq. (2), (2) adding random group effects ${G}_{z}$ in Eq. (1) (with standard deviation ${\upsigma }_{\mathrm{G}}$), (3) adding a fixed effect (e.g. $\mathbf{X}{\mathbf{b}}_{{\varvec{g}},{\varvec{f}},{\varvec{r}},}$) in Eq. (2),^23^ (4) analysing data with unknown infection times, and (5) assuming only periodic disease status checks on individuals. Results of this investigation (for details see Additional file 11) showed that, while statistical power was reduced (by varying amounts), the optimal design features illustrated in Fig. 2 remained (approximately) unchanged.^24^

**Fig. 6:**
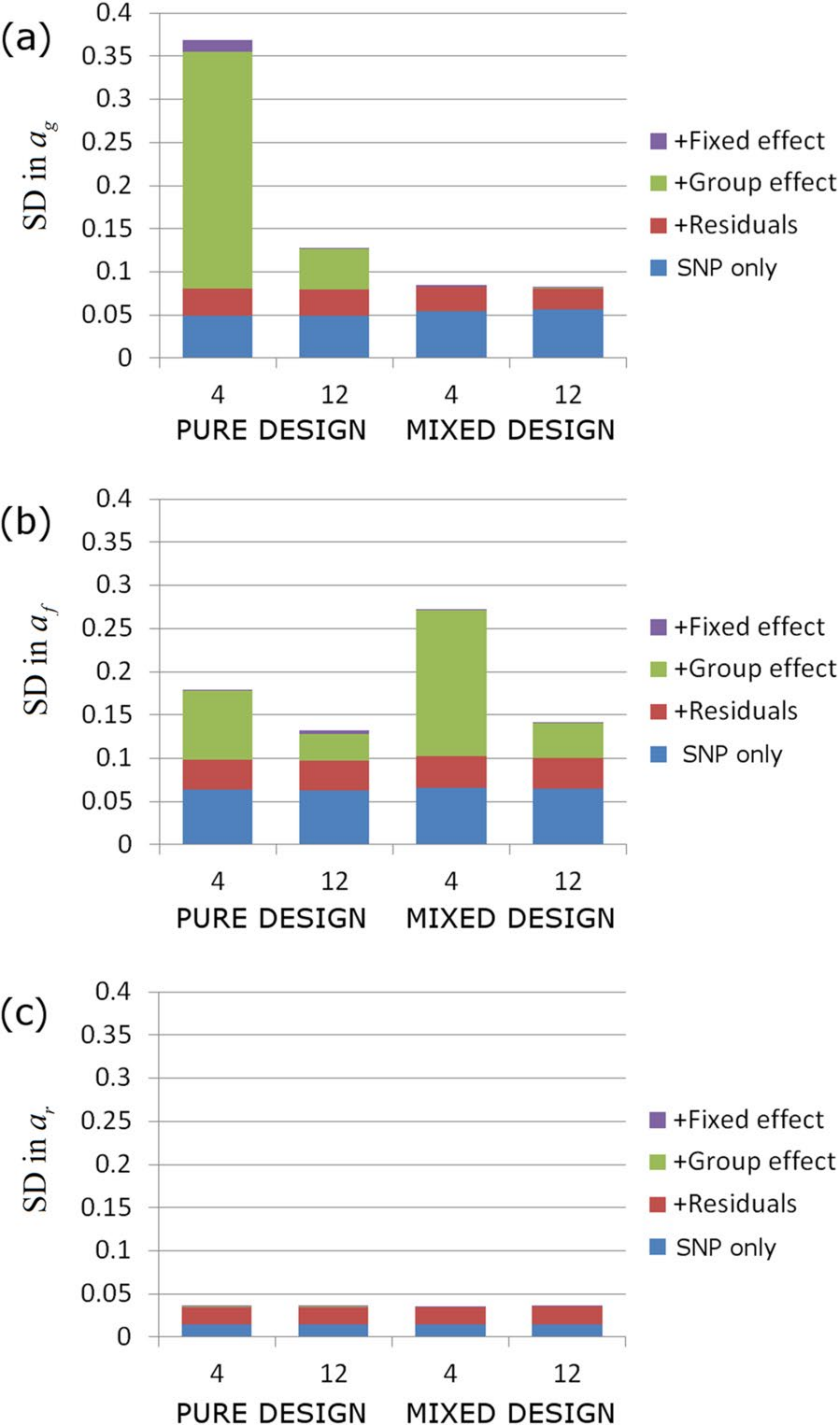
Partitioning contributions to standard deviations for estimates of SNP effects. Residuals, group effects and a fixed effect are sequentially added to the basic SNP-only model (infection and recovery times assumed known). The corresponding increase in the SDs in the posterior distributions for SNP effects is investigated for (**a**) the susceptibility ${a}_{g}$, **b** the infectivity ${a}_{f}$, and **c** the recoverability ${a}_{r}$. For comparison, four different scenarios are investigated: a pure design (no dominance estimate) with respectively ${N}_{\mathrm{group}}=4$ (i.e. a single replicate of the basic design) and ${N}_{\mathrm{group}}=12$ (i.e. three replicates of the basic design) and a mixed design (no dominance estimate) with ${N}_{\mathrm{group}}=4$ (i.e. two replicates) and ${N}_{\mathrm{group}}=4$ (i.e. six replicates). In each case, ~ 1000 individuals were partitioned equally among the contact groups. The residuals were chosen to have the covariance matrix ${{\varvec{\Sigma}}}_{gg}={{\varvec{\Sigma}}}_{ff}={{\varvec{\Sigma}}}_{rr}=1$, ${{\varvec{\Sigma}}}_{gf}=0.3$, ${{\varvec{\Sigma}}}_{gr}=-0.4$, and ${{\varvec{\Sigma}}}_{fr}=-0.2$, the group effects had a SD of ${\upsigma }_{G}=0.2$, and the fixed effect (assumed to represent sex with gender randomly allocated) had a size ${b}_{g0}={b}_{f0}={b}_{r0}=0.2$. Results were found to be largely insensitive to these essentially arbitrary choices

In the following, we sequentially add residual (${\varvec{\upvarepsilon}}$ in Eq. (2)), group (${G}_{z}$ in Eq. (1)), and fixed effect contributions ($\mathbf{X}{\mathbf{b}}_{{\varvec{g}},{\varvec{f}},{\varvec{r}},}$ in Eq. (1)) to the basic SNP-only model (i.e. the model without any of these other effects) to evaluate how this impacts the precision of SNP effect estimates. Focusing on the optimal pure and mixed designs (with no dominance estimate), results are shown in Fig. 6. These analyses assume a fixed total number of individuals ${N}_{\mathrm{total}}$ = 1000 and considers cases with 4 and 12 (through replication) contact groups (for results with only 100 individuals see Additional file 12). The following conclusions can be drawn: (1) the SD for ${a}_{r}$ are smallest (and least affected by additional sources of variation), followed by the SD for ${a}_{g}$, then the SD for ${a}_{f}$; (2) the SD for the basic SNP-only model (i.e*.* without group, fixed or residual effects in Eqs. (1) and (2)) and for the model when residual effects are added is largely independent of the choice of design (pure or mixed) or of the number of replicates; (3) the increase in SDs when a group effect is added to the model is substantially smaller when the number of contact groups is increased from 4 to 12, and falls towards zero as the number of contact groups becomes larger (see Additional file 13: Fig. S10); (4) as shown in Fig. 6a, the group effect causes a big increase in the SD in ${a}_{g}$ for the pure model but had almost no effect for the mixed model (this is because in the pure model the contacts are genetically homogenous, so estimation of their susceptibility becomes confounded with the group effect, whereas in the mixed design, the relative infection times of individuals of different genotypes provides direct information for their relative susceptibility, irrespective of the group effect); (5) as shown in Fig. 6b, the group effect provides a slightly larger increase in the SD in ${a}_{f}$ for the mixed design compared to the pure design; and (6) adding fixed effects to the model leads to very little change in the SNP effect SDs, provided they are not substantially correlated with the genotype of individuals (see Additional file 14: Fig. S11).

### Design tool software

**Fig. 7:**
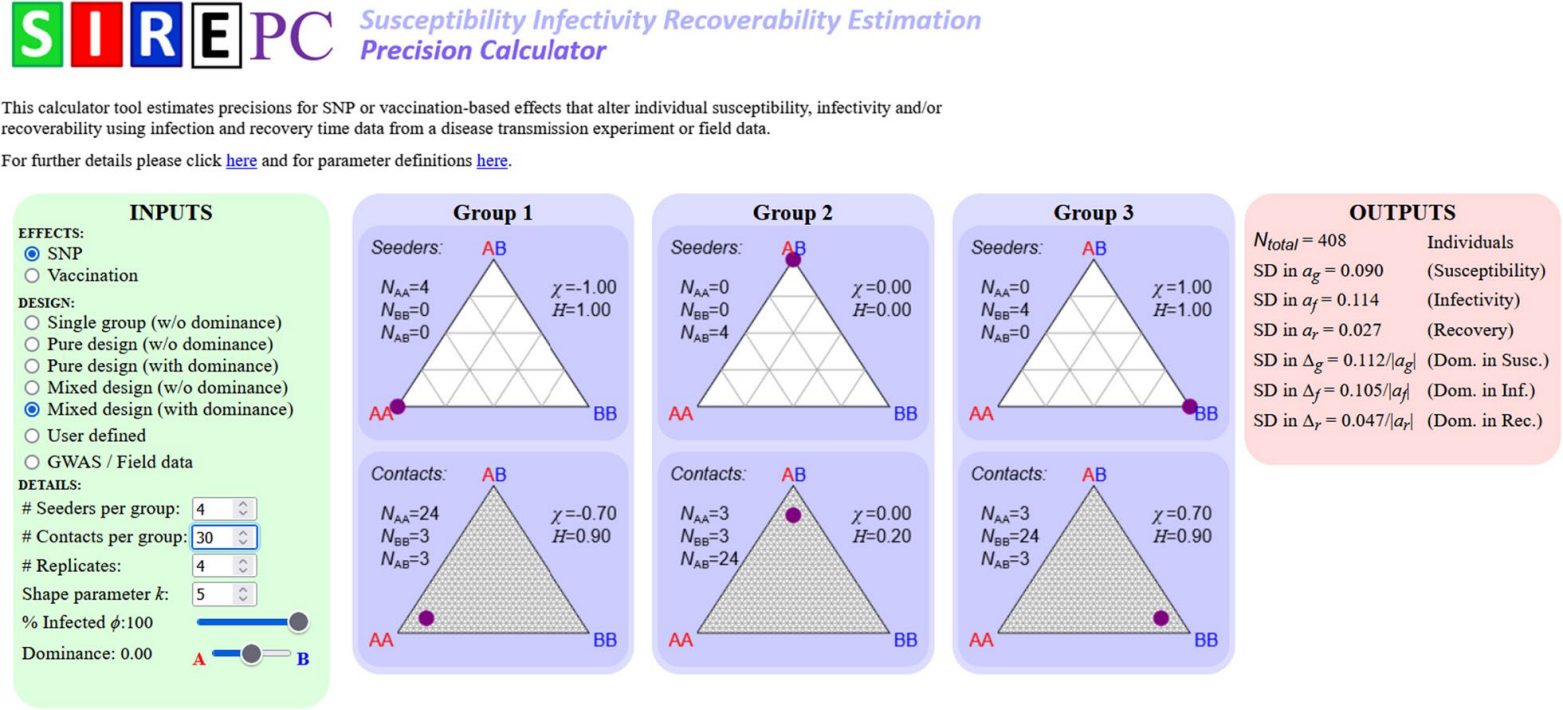
Precision Calculator tool*.* SIRE-PC (susceptibility infectivity recoverability estimation precision calculator) is an easy-to-use online software that calculates the analytical expressions provided in the "Results" section to help aid experimental design

The analytical expressions derived in this study, together with the diverse experimental designs, were implemented in the user-friendly online software tool SIRE-PC (susceptibility infectivity recoverability estimation precision calculator) (Fig. 7). This calculator takes details of the experimental design as user inputs, specifically the number and genetic composition of seeders and contacts in each group, the number of replicates, an estimate for the fraction of contacts expected to become infected ($\phi )$, and the shape parameter that characterizes dispersion in recovery times ($k$). The outputs generated are the total number of individuals used in the experiment and analytical estimates for the SD in SNP effects ${a}_{g}$, ${a}_{f}$, and ${a}_{r}$ in Eqs. (6) to (8), and for ${\Delta }_{g}$, ${\Delta }_{f}$ and ${\Delta }_{r}$ in Eqs. (10), (11), and (13). Note that these expressions only consider the complete and no dominance cases, but the software actually allows intermediate dominance to be investigated as well.

Determining the appropriate experimental design is achieved by adjusting the input values, subject to any practical/logistic limitations (e.g. the number individuals per contact group may be fixed), with the aim of minimising the SD in the SNP effects. To facilitate this process, the tool includes the optimal experimental designs in Fig. 2 and also provides the option for arbitrary user-defined designs to be investigated. Moreover, the software allows for precision estimates when studying vaccination effects on the three host epidemiological traits, as well as applications to GWAS and field data, as explained in the “Discussion” section.

## Discussion

There has been increasing acknowledgment within the livestock genetics community that the spread of infectious disease in populations may not only depend on the genetic susceptibility of individuals to infection but also on their genetic infectivity and recoverability [25, 29, 31, 32]. Indeed, Tsairidou et al*.* [27] showed the importance of selecting for reduced infectivity in genetic disease control due to the large expected variation in this trait. While methods for estimating effects of SNPs and other genetic effects for these novel host traits from epidemiological data are emerging [15, 28–30], to date, limited consideration has been given to the optimal design of transmission experiments. This study demonstrates that considerable improvements in the precision of estimates of SNP effects associated with all three host epidemiological traits can be achieved by choosing the appropriate experimental design. Here, this is explicitly illustrated by means of considering a single SNP with potential effects on all three host epidemiological traits, but the same basic design features apply to any other categorical fixed effect (e.g. sex, family, line, vaccination status of individuals).

This study provides analytical expressions for the precision of estimated SNP substitution and dominance effects associated with host susceptibility, infectivity, and recoverability, which have been implemented in an online software tool to assist with the design of transmission experiments and for statistical power analyses in experimental studies. To make the derivations tractable, the calculations were shown for a best-case scenario, in which non-SNP contributions were ignored and infection and recovery times were assumed known. Nonetheless, the derived expressions were found to be in strong agreement with numerical results obtained by performing inference on data from simulated epidemics that account for a range of complications and confounding that are likely to be present in real data. The parameter that was found to be most difficult to precisely estimate was ${a}_{f}$, which characterises differences in infectivity (as a result of it being an indirect effect). In contrast, estimates for susceptibility and recoverability effects were found to be relatively insensitive to the experimental design. Consequently, optimizing experimental designs largely focused on improving the precision of ${a}_{f}$ estimates.

From these analyses, three types of optimal designs emerged. The first of these considered just a single contact group. It was found that, in principle at least, it is possible to infer ${a}_{f}$ if the group contains enough individuals. However, this would not be a recommended option because multiple groups provide a way of significantly increasing statistical power (as they allow for a direct comparison of epidemic behaviour between groups with substantially different genetic makeup). When implementing designs with multiple groups, two fundamentally different strategies were found: the “pure” and the “mixed” designs. The pure design uses seeders and contacts which, within themselves, have the same SNP genotype, but with seeder and contact genotypes permutede across different contact groups, as shown in Fig. 2b (in cases in which dominance is not being investigated this consists of replicates of four groups, and when it is, replicates of nine groups). Choosing an approximately equal number of seeders and contacts led to similar precisions for estimates of SNP effects on susceptibility and infectivity. In contrast, the mixed design relies on different frequencies of genotypes in the contact population across groups, as shown in Fig. 2c, with just a few seeders^25^ (here replicates of two or three groups are needed, depending on whether dominance is being investigated or not).

For a fixed total number of individuals, both the pure and mixed designs were found to be similar in terms of their precision for estimating SNP effects (see Table 3). However two features of the mixed design make it advantageous over the pure design: (1) when group effects are included, it was found to be significantly better at estimating SNP effects on susceptibility^26^ (because differences in infection times of individuals of different genotypes provide direct evidence for differences in susceptibility, irrespective of group effect), and (2) it requires far fewer seeders. The latter is particularly important for disease transmission experiments in which seeders are artificially infected and, therefore, may behave differently than contacts, who naturally acquire infection during the experiment.^27^ Consequently, we advocate the mixed design as the best approach to take because it largely relies on information from contacts, sidestepping the difficult issue of whether artificially infected individuals are epidemiologically representative of natural infections. Interestingly, this design is much less intuitive^28^ than the pure design, illustrating the importance of the analytical expressions derived in this work.

When implementing optimal multiple group designs, the analytical expressions in Table 3 suggest that the precision of parameter estimates is largely independent of the number of individuals within a group, given a fixed total (for example, using four groups in the pure design in Fig. 2b gave a very similar level of precision to using two replicates of four groups, each containing half the number of individuals). However in the more realistic scenario of significant random differences in disease transmission between groups (necessitating incorporation of the group effect term ${G}_{z}$ in Eq. (1)), more replicates with smaller groups was found to be beneficial (especially true for the estimate of the SNP effect on infectivity).^29^

To the best of our knowledge, to date relatively few animal disease transmission experiments have been conducted to specifically estimate host genetic effects on epidemiological traits [25, 34, 35]. Due to logistic restrictions on the number of contact groups, a multi-group pure design was used in a recent experiment involving infectious salmon anemia virus transmission in Atlantic salmon [34]. Although the experiment lacked statistical power to provide precise estimates for genetic effects, it revealed that high genetic resistance may not necessarily confer beneficial effects on the epidemiological traits, as previously indicated for resistance of Atlantic salmon to the IPN virus [25]. Similar findings were reported in a recent small-scale porcine reproductive and respiratory syndrome (PRRS) virus transmission experiment in pigs to assess the effects of the previously identified *GBP5* PRRS resistance gene on pigs’ susceptibility and infectivity under natural conditions [34, 36]. That experiment adopted a multi-group mixed design, but also used barcoding of the virus to track pig genotype-specific transmission routes in order to increase statistical power. A multi-group mixed design was also adopted in a larger transmission experiment that aimed at estimating family effects on all three host epidemiological traits for parasite (*Philasterides dicentrarchi*) infections in turbot fish [35]. The design of this experiment was guided by earlier studies on optimising estimates of indirect genetic effects (such as infectivity), which advocated designs with two or more families per contact group [37, 38].

Many previous studies have investigated the effects of vaccines on disease transmission in farmed animals (see e.g. [8] for a review), and corresponding optimal experimental designs [39, 40]. However, only relatively few transmission experiments explicitly distinguish between the direct effects of vaccines on host susceptibility and their indirect effects on host infectivity. Van der Goot et al*.* [41] used multi-group pure designs with an equal number of seeders and contact individuals (identified as the optimal ratio in our study) to estimate vaccine effects on the epidemiological host traits for avian influenza in chicken. However, in line with our results, a previous simulation study also identified a multi-group mixed design with a varying fraction of vaccinated susceptible individuals across contact groups as optimal for simultaneously estimating vaccine effects on host susceptibility and infectiousness parameters [40]. In that study, the optimal fraction of vaccinated individuals in each contact group depended on the effect size of the vaccine, on the epidemiological traits under consideration, and on the basic reproductive ratio for estimating infectivity effects. Based on our analytical expressions, the effect size only affected the precision of dominance effects, while the basic reproductive ratio had little effect on precision.

### Implications for genome-wide association studies

This study investigated experimental designs for which the composition of the seeder and contact populations were tailored to estimate the effects of a specific SNP of interest on all three host epidemiological traits. However, suppose that we are interested in performing a genome-wide association study (GWAS). In this case, allocation of seeders and contacts according to their SNP genotype is not possible (because the genotype composition will be different for each SNP). So how should experiments be optimally designed in this case? An analysis based on considering an arbitrary SNP that is in Hardy–Weinberg equilibrium [33], with frequency $p$ of allele *A* in a population consisting of unrelated individuals is in Additional file 15. The results showed that, as with the mixed design, precisions of estimates of SNP effects are maximised when epidemics are instigated with few seeders, and the following results can be derived:

$${\mathrm{SD\,in\,}a}_{g}\cong \frac{1}{\sqrt{2p\left(1-p\right){N}_{\mathrm{total}}}},$$

$${\mathrm{SD\,in\,}a}_{f}\cong \frac{1}{\sqrt{2p\left(1-p\right)\left(2-\frac{1}{{G}_{\mathrm{size}}-1}\right){N}_{\mathrm{group}}}},$$

17 $$\mathrm{SD\,in\,}{a}_{r}\cong \frac{1}{\sqrt{2p\left(1-p\right){kN}_{\mathrm{total}}}}.$$

Note here that these SDs crucially depend on $p$, which makes sense in the limits $p\to 0$ and $p\to 1$, as the population becomes uniformly homozygous with no information regarding SNP effects. Most important, compared to the results in Table 3, the SD in ${a}_{f}$ now contains ${N}_{\mathrm{group}}$ in the denominator instead of ${N}_{\mathrm{total}}$. This means that increasing the number of individuals in each contact group no longer substantially increases the precision with which ${a}_{f}$ can be estimated (a feature noted in [15]). Consequently, when performing GWAS, many contact groups with fewer individuals lead to greater precision in estimating the SNP effect for infectivity (which, interestingly, is not the case for the susceptibility or recoverability effects). This hinges on the fact that infectivity acts on other individuals in the group, so smaller groups allow for more information regarding who is infecting whom. Although the derivations were based on genetically unrelated individuals, these observations are expected to remain valid for genetically structured populations.

### Field data

We now consider the possibilities and additional complications that arise when considering field data (that is data obtained from real-world disease outbreaks). As with the GWAS discussion above, here we do not have the luxury of being able to choose the composition of groups in terms of SNP genotypes. Nevertheless, the analytical expression in Eq. (17) provide power calculations that can estimate what could, in principle, be inferred (and again point to the fact that smaller groups sizes are more likely to yield good estimates for infectivity SNP effects). In the case of field data, the “seeders” are “index” cases which instigate the epidemics. Fortunately, the fact that there is usually just one index case coincides with the optimum for the precision of estimates of SNP effects, as discussed above.

When investigating vaccination effects, presence of some groups with a high vaccination rate and others with a low (or no) vaccination rate, would naturally lend itself to something akin to the optimal mixed design proposed in this paper. Hence, we would expect such experimental vaccination designs to be highly informative not only about susceptibility and recoverability effects, but also about infectivity.

It should be mentioned that analysis of real-world data comes with additional complications: (1) proper accounting for related individuals; (2) the fact that groups are not entirely closed (e.g. cows in different fields may share milking facilities); and (3) not all individuals start in the susceptible state, especially for endemic diseases. Tackling these problems will require further development of the approaches outlined in this paper.

### Further considerations

In this paper, epidemics were modelled using SIR dynamics, but it is important to point out that the results are equally applicable to diseases for which individuals do not recover (i.e. the SI model). In these cases, estimates of SNP effects on susceptibility and infectivity can be used in selective breeding programs to reduce disease prevalence. Although more complicated compartmental models were not investigated, e.g. the inclusion of an exposed (infected but not infectious) state, the basic idea of accentuating differences between contact groups for the factor under study (e.g. by ensuring large differences in the SNP genotypic composition in the mixed and pure designs) to increase variation in epidemic speed (which in turn provides evidence for variation in infectivity), is expected to remain valid.

Table 3 provides a useful guide as to the size of the SNP effects that can be detected from a given experiment. It suggests that for datasets comprising 1000 individuals or fewer, only SNPs with large effects (typically explaining more than 15% of the total phenotypic variation) on the epidemiological host traits can be accurately estimated. Detection of the effects of SNPs with small to moderate effects would require significantly more data, in particular for infectivity. Although potentially challenging for livestock species due to the cost, such large-scale experiments may be feasible for aquaculture and for smaller laboratory species (e.g*.* insects).

Although SNPs with large effects on disease resistance have been identified [11, 22, 42], there is evidence to suggest that disease resistance is mostly polygenic [43]. So far, little is known about the genetic architecture underlying host infectivity and recoverability, but it seems reasonable to expect that these may also be mostly under polygenic regulation. This study has ignored any polygenic contributions to Eq. (2) [28] by assuming that members from different families are distributed randomly across groups. Incorporation of such effects may lead to new insights into optimal disease transmission design, and will be the subject of future research.

## Conclusions

The aim of this paper was to identify optimal designs for disease transmission experiments to estimate the effects of a particular SNP of interest (or other factors) on the susceptibility, infectivity, and recoverability of individuals. It was found that while the precision of estimates of susceptibility and recoverability effect were relatively insensitive to the design for a given total number of contact individuals (both being clearly related to the infection and recovery times of individuals themselves), infectivity was not (because its effects are evident from epidemiological data of other individuals). In particular, to precisely estimate genetic effects on infectivity, a so-called “mixed” design was identified, which specifies the optimal proportions of different genotypes in the contact populations of different groups. Replication of this basic design was found to be effective at reducing confounding that can arise from group effects. An easy-to-use software tool accompanying this paper was developed to aid experimental design by providing estimates for the precision of parameter estimates. The results shown here illustrate that such estimates are reliable and robust to noise and to a range of potential confounding factors that are likely present in real-world disease systems.

## Supplementary Information

**Additional file 1:** Provides details on how inference was performed on simulated datasets to generate results against which the analytical expressions could be compared.

**Additional file 2: **Derivation of the observed Fisher information matrix [45–47]. Description: This section derives analytical expressions for the matrix in Eq. (4).

**Additional file 3: **Inversion of the observed Fisher information matrix. Description: This shows how the observed Fisher information matrix in Eq. (4) is inverted to give the results in Eqs. (6) and (7).

**Additional file 4: **Derivation of standard deviations for recoverability SNP effect. Description: The standard deviation for the SNP effect on recoverability* a*_*r*_ is derived.

**Additional file 5: **Derivation of standard deviations in the dominance parameters Δ_*g*_, Δ_*f*_ and Δ_*r*_.

**Additional file 6: Fig. S1**. Shows how the probability of epidemic extinction varies as a function of the number of seeder individuals* N*_seed_ for different basic reproductive ratios* R*_0_.

**Additional file 7: **Further details for the single contact group design. Description: Provides analytical expressions for the standard deviations in the SNP effect parameters *a*_*g*_, *a*_*f*_ and *a*_*r*_ for the single contact group design.

**Additional file 8: Fig. S2**. Impact of changing* R*_0_. In additional file 8, we numerically investigate how changing* R*_0_ affects the fraction of infected contacts* ϕ* and the optimal design choices outlined in Fig. 2.

**Additional file 9: Fig. S3**. Further details for the “pure” design. Description: Provides analytical expressions for the standard deviations in the SNP effect parameters *a*_*g*_, *a*_*f*_ and *a*_*r*_ and dominance parameters Δ_*g*_, Δ_*f*_ and Δ_*r*_ for the “pure” design.

**Additional file 10: Fig. S4**. Further details for the “mixed” design. Description: Provides analytical expressions for the standard deviations in the SNP effect parameters *a*_*g*_, *a*_*f*_ and *a*_*r*_ and dominance parameters Δ_*g*_, Δ_*f*_ and Δ_*r*_ for the “mixed” design.

**Additional file 11: **Impact of realistic model/data scenarios on design. Description: Considering the optimal designs in Fig. 2, this additional file investigates the impact of separately introducing five additional sources of variation into the model/data (which, for the purposes of analysis, were ignored).** Fig. S5**. Impact of sources of model/data variation on optimal design for infectivity.** Fig. S6**. Impact of sources of model/data variation on optimal design for susceptibility.** Fig. S7**. Impact of sources of model/data variation on optimal design for recoverability.** Fig. S8**. Impact of residual contributions.

**Additional file 12: Fig. S9**. Partitioning contributions to the SD of SNP effects. Description: Figure 6 in the paper shows the result of sequentially adding residuals, group effects and a fixed effect to the basic SNP-only model. Here, we present the corresponding results assuming only 100 individuals.

**Additional file 13: Fig. S10**. Design replication. Description: Investigates how the reduction in precision when incorporating group effects can be moderated by means of design replication (that is repeating the same basic designs in Fig. 2 several times).

**Additional file 14: Fig. S11**. Figure S11 investigates the addition of a single large fixed effect with elements in the design matrix** X** set in such a way as to give a certain degree of correlation with the SNP.

**Additional file 15**: Rather than defining the proportions of genotypes in the seeder and contact populations, we consider the case in which individual genotypes are randomly allocated with *A* allele having frequency* p*, assuming Hardy-Weinberg equilibrium.** Fig. S12**. Precision estimates for the SNP parameters with random genotype allocation.

## Data Availability

All data generated or analysed during this study are included in this published article and its Additional files.

## Footnotes

1 It should be mentioned that the methodology and design principles outlined in this paper assume that ${R}_{0}$ is higher than 1 for each contact group, so allowing for sustained disease propagation. This may not be the case, e.g., for effective vaccines that confer a drastically reduced transmission rate.

2 Providing degradation of the pathogen in the environment is relatively fast, i.e. accumulation is not accounted for.

3 In the case of field data these would be index cases.

4 From an epidemiological standpoint these are equivalent because they both remove the infected individual from the system.

5 E.g. ${\chi }_{\mathrm{seed}}=1$ if seeders are only *AA* individuals, ${\chi }_{\mathrm{seed}}=-1$ if only *BB* individuals, and somewhere in between in the general case.

6 “Fractional deviation” is defined by the exponential dependency in Eq. (1), e.g. ${g}_{j}=0.1$ corresponds to individual $j$ being a fraction $\approx$ 10% more susceptible than a population-wide reference.

7 Over and above those coming from the SNP and fixed effects, themselves.

8 Marginalised means that all parameters other than the one being considered are integrated out of the likelihood, so leaving a probability distribution for that parameter. The mean of this distribution can be used as a parameter estimate and the standard deviation characterises the precision of that estimate.

9 In the limit of large basic reproductive ratio *R*_*0*_, $\phi$ is 1, but for *R*_*0*_ close to 1, *ϕ* may be substantially smaller (see Additional file 8 for details).

10 *E.g*., if infections happen quickly in groups with seeders that have a high proportion of *A* alleles (irrespective of the genotype of the contacts), this provides direct evidence that *A* alleles confer greater infectivity.

11 This scaling is familiar in many statistical models where estimated contrasts typically scale with $1/\sqrt{N}$, where $N$ is the number of observations, simply because variances of averages scale with $1/N$.

12 E.g. in the case of livestock, pens are usually designed to house a certain number of animals.

13 Importantly, optimisation was not found to detrimentally affect the precision of susceptibility and recoverability effects, which are themselves found to be relatively insensitive to experimental design.

14 Although not proven, numerical investigations suggest that experimental designs that have significant contributions from both terms in the denominator of Eq. (7) are no better than those shown in Fig. 2.

15 In the continuum limit, the fraction of individuals that become infected $\phi$ is given by the solution of the transcendental equation $\phi =1-{e}^{{-R}_{0}\phi }$, e.g. $\phi$ = 0.94 for ${R}_{0}$ = 3 and $\phi$ rapidly approaches 1 for higher ${R}_{0}$. See Additional file 8: Fig. S1 for a numerical solution to this equation.

16 Reduction of $\phi$ below 1 means that the analytical expressions in Eqs. (14) to (16) marginally overestimate precision.

17 Here seeders are set to be *BB* individuals, corresponding to a homozygote balance of ${\chi }_{\mathrm{seed}}$ = − 1, and contacts are set to be 70% *AA* and 30% *BB*, corresponding to ${\chi }_{\mathrm{cont}}$ = 0.7 − 0.3 = 0.4. Note, the trend for fewer seeders leading to higher precision in Fig. 3a is true for any ${\chi }_{\mathrm{cont}}$.

18 If they are identical, the genetic makeup of infected individuals remains approximately unchanged as the epidemic progresses and infectivity and susceptibility effects become confounded.

19 Note here that relative “speed” has to explicitly take the number of infected individuals into account, as defined by the model.

20 Importantly, the genetic composition in the contact population does *not* change (in the example it remains solely *AA*), and so there is no confounding between susceptibility and infectivity.

21 Seeders all have the same genotype and contacts all have the same genotype, but seeders and contacts may have different genotypes.

22 Consideration of the small first term in the denominator of Eq. (7) shows that the seeder population is actually optimised when there are solely *AA* seeders in group 1 and solely *BB* seeders in group 2.

23 Specifically $\mathbf{X}$ is a vector with elements randomly selected to be + 0.5 and − 0.5 representing male/female and ${b}_{g0}$, ${b}_{f0}$, ${b}_{r0}$ are fixed effects that characterise how sex affects the three traits.

24 The only exception to this was that under the pure design the optimal fraction of seeders reduced to around 20% when group effects were introduced, but this increased back towards the optimal 47% with repeated experimental replicates.

25 Typically two or three, sufficiently large to avoid the problem of epidemic extinction.

26 Although there is a corresponding small reduction in the precision for infectivity SNP effects.

27 This can be mitigated by the so-called extended experimental design (in which artificially infected individuals are used to infect seeders prior to the start of the experiment), however, this not only increases the cost of the experiment, but also introduces additional uncertainty in the infection times of seeders which needs to be accounted for.

28 Intuitive in the sense that the pure design can be argued based on the fact that seeders and contacts provide information about infectivity and susceptibility, respectively, so they should each consist of a similar number of individuals and different groups should go through the genotypic combinations for each. On the other hand, deriving the optimal genotype fractions for the mixed design is not intuitively obvious.

29 In the case of a single replicate there is often confounding as to whether the overall speed of an epidemic within a group is due to its genetic makeup or the group effect which acts on the transmission rate. However, if several replicates exhibit a similar behaviour (i.e. they are all slow) this directly points to the genetic effect (group effects average to zero because they are uncorrelated across groups).
