# Supplementary material for: Optimal experimental designs for estimating genetic and non-genetic effects underlying infectious disease transmission
Source: Genet Sel Evol. 2022 Sep 5;54:59. doi: 10.1186/s12711-022-00747-1 (PMC9442948; doi:10.1186/s12711-022-00747-1)
Supplement: Supplementary file 3 — Additional file 3: Inversion of the observed Fisher information matrix. Description: This shows how the observed Fisher information matrix in Eq. (4) is inverted to give the results in Eqs. (6) and (7). [file 12711_2022_747_MOESM3_ESM.pdf]

## Inversion of the observed Fisher information matrix

The inverse of the Fisher information matrix is given by

$$\mathbf{M}^{-1} = \frac{1}{M_{gg}M_{ff} - M_{gf}^2}\begin{pmatrix} M_{ff} & -M_{gf} \\ -M_{gf} & M_{gg} \end{pmatrix}. \tag{A1}$$

The diagonals of this matrix give the inverse of the marginalised variances in $a_g$ and $a_f$:

$$\begin{aligned} \frac{1}{\mathrm{Var}(a_g)} &= M_{gg} - \frac{M_{gf}^2}{M_{ff}}, \\ \frac{1}{\mathrm{Var}(a_f)} &= M_{ff} - \frac{M_{gf}^2}{M_{gg}}. \end{aligned} \tag{A2}$$

Substituting in terms from the matrix in Eq.(4) gives

$$\begin{aligned} \frac{1}{\mathrm{Var}(a_g)} &= N_{\text{group}}\phi N_{\text{cont}}\left(\langle H_{\text{cont}}\rangle - \langle \chi_{\text{cont}}\rangle^2\right) - \frac{\left(N_{\text{group}}\phi N_{\text{cont}}\mathrm{Var}(\chi_{\text{cont}}) + N_{\text{group}}N_{\text{seed}}W\right)^2}{N_{\text{group}}\phi N_{\text{cont}}\mathrm{Var}(\chi_{\text{cont}}) + N_{\text{group}}N_{\text{seed}}(2W+Y)}, \\ \frac{1}{\mathrm{Var}(a_f)} &= N_{\text{group}}\phi N_{\text{cont}}\mathrm{Var}(\chi_{\text{cont}}) + N_{\text{group}}N_{\text{seed}}(2W+Y) - \frac{\left(N_{\text{group}}\phi N_{\text{cont}}\mathrm{Var}(\chi_{\text{cont}}) + N_{\text{group}}N_{\text{seed}}W\right)^2}{N_{\text{group}}\phi N_{\text{cont}}\left(\langle H_{\text{cont}}\rangle - \langle \chi_{\text{cont}}\rangle^2\right)}. \end{aligned}$$

(A3)

The first expression can be rearranged in the following way:

$$\begin{aligned} \frac{1}{\mathrm{Var}(a_g)} &= N_{\text{group}}\phi N_{\text{cont}}\left(\langle H_{\text{cont}}\rangle - \langle \chi_{\text{cont}}^2\rangle\right) + N_{\text{group}}N_{\text{seed}}\frac{\phi N_{\text{cont}}\mathrm{Var}(\chi_{\text{cont}})Y - N_{\text{seed}}W^2}{\phi N_{\text{cont}}\mathrm{Var}(\chi_{\text{cont}}) + N_{\text{seed}}(2W+Y)}, \\ &= N_{\text{group}}\phi N_{\text{cont}}\left(\langle H_{\text{cont}}\rangle - \langle \chi_{\text{cont}}^2\rangle\right) + N_{\text{group}}N_{\text{seed}}\left[Y - \frac{N_{\text{seed}}(W+Y)^2}{\phi N_{\text{cont}}\mathrm{Var}(\chi_{\text{cont}}) + N_{\text{seed}}(2W+Y)}\right], \end{aligned}$$

(A4)

as shown in Eq.(6). The expression for $\mathrm{Var}(a_f)$ from Eq.(A3) can be written

$$\frac{1}{\mathrm{Var}(a_f)} = N_{\text{group}} N_{\text{seed}} Y + \frac{\left(N_{\text{group}}\phi N_{\text{cont}} \mathrm{Var}\left(\chi_{\text{cont}}\right) + 2N_{\text{group}} N_{\text{seed}} W\right)\left(\left\langle H_{\text{cont}}\right\rangle - \left\langle \chi_{\text{cont}}\right\rangle^2\right) - \frac{\left(N_{\text{group}}\phi N_{\text{cont}} \mathrm{Var}\left(\chi_{\text{cont}}\right) + N_{\text{group}} N_{\text{seed}} W\right)^2}{N_{\text{group}}\phi N_{\text{cont}}}}{\left\langle H_{\text{cont}}\right\rangle - \left\langle \chi_{\text{cont}}\right\rangle^2},$$

$$= N_{\text{group}} N_{\text{seed}} Y + \frac{\left(N_{\text{group}}\phi N_{\text{cont}} \mathrm{Var}\left(\chi_{\text{cont}}\right) + 2N_{\text{group}} N_{\text{seed}} W\right)\left(\left\langle H_{\text{cont}}\right\rangle - \left\langle \chi_{\text{cont}}^2\right\rangle\right) - \frac{N_{\text{seed}}^2}{\phi N_{\text{cont}}} N_{\text{group}} W^2}{\left\langle H_{\text{cont}}\right\rangle - \left\langle \chi_{\text{cont}}\right\rangle^2},$$

$$= N_{\text{group}} N_{\text{seed}} \left[ Y + \frac{2W\left(\left\langle H_{\text{cont}}\right\rangle - \left\langle \chi_{\text{cont}}^2\right\rangle\right) - \frac{N_{\text{seed}}}{\phi N_{\text{cont}}} W^2}{\left\langle H_{\text{cont}}\right\rangle - \left\langle \chi_{\text{cont}}\right\rangle^2} \right] + N_{\text{group}}\phi N_{\text{cont}} \frac{\left\langle H_{\text{cont}}\right\rangle - \left\langle \chi_{\text{cont}}^2\right\rangle}{\left\langle H_{\text{cont}}\right\rangle - \left\langle \chi_{\text{cont}}\right\rangle^2} \mathrm{Var}(\chi_{\text{cont}}),$$

(A5)

which is given in Eq.(7).
