# Supplementary material for: Optimal experimental designs for estimating genetic and non-genetic effects underlying infectious disease transmission
Source: Genet Sel Evol. 2022 Sep 5;54:59. doi: 10.1186/s12711-022-00747-1 (PMC9442948; doi:10.1186/s12711-022-00747-1)
Supplement: Supplementary file 7 — Additional file 7: Further details for the single contact group design. Description: Provides analytical expressions for the standard deviations in the SNP effect parameters ag, af and ar for the single contact group design. [file 12711_2022_747_MOESM7_ESM.pdf]

Further details for the single contact group design

Under the single contact group design in Fig. 2a, the SDs in the SNP effects are given by:

$$\text{SD in } a_g \cong \frac{1}{\sqrt{N_{\text{total}}(1-h)}},$$
$$\text{SD in } a_f \cong \frac{1}{\sqrt{N_{\text{total}}\left(h(1-h)-\frac{h^2}{1-h}\log^2(h)\right)(\chi_{\text{seed}}-\chi_{\text{cont}})^2}}, \quad \text{(A1)}$$
$$\text{SD in } a_r = \frac{1}{\sqrt{kN_{\text{total}}}},$$

where $h=N_{\text{seed}}/G_{\text{size}}$ is the fraction of seeder individuals (here contacts are all assumed to become infected, *i.e.* $\phi\approx1$, appropriate for reasonably large R_0).

Since this design contains no heterozygote *AB* individuals, no information is available for estimating the dominance parameters. The expressions reported in Table 2 come from substituting the optimal design values from Fig. 2a into Eq.(A1).
