# Supplementary material for: Optimal experimental designs for estimating genetic and non-genetic effects underlying infectious disease transmission
Source: Genet Sel Evol. 2022 Sep 5;54:59. doi: 10.1186/s12711-022-00747-1 (PMC9442948; doi:10.1186/s12711-022-00747-1)
Supplement: Supplementary file 8 — Additional file 8: Fig. S2. Impact of changing R0. In additional file 8, we numerically investigate how changing R0 affects the fraction of infected contacts ϕ and the optimal design choices outlined in Fig. 2. [file 12711_2022_747_MOESM8_ESM.pdf]

Impact of changing R_0

As the basic reproductive ratio R_0 becomes closer to one, so the fraction of contact individuals that become infected during experiments is expected to go down. This additional file numerically investigates how this affects the optimal design choices outlined in Fig. 2.

For each experimental design different values of R_0 were chosen (by varying β and fixing γ). A large number of simulations were performed (10^5) to establish the average fraction of contacts ϕ which become infected. The SD in a_f was estimated using the analytical expression from Eq.(7). The procedure was repeated whilst varying critical design parameters (*e.g.* the fraction of individual which are seeders in the case of the single contact group). The parameter value that minimised the SD in a_f is plotted in Fig. S2 (the five sub-plots correspond to the designs in Fig. 2). We observe that all the optimal designs are largely insensitive to the exact value of R_0.

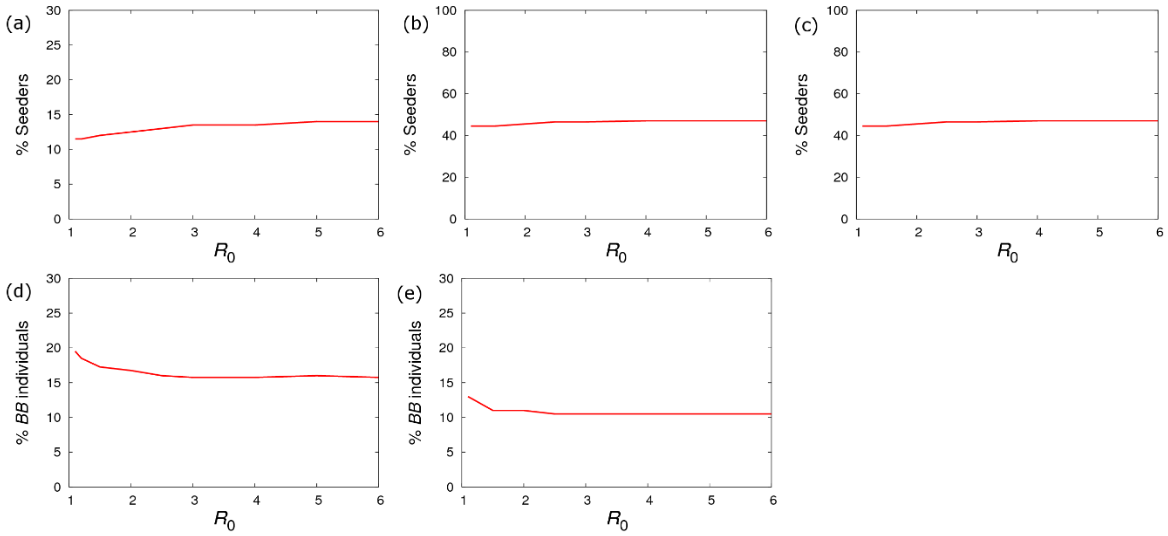

Figure S2. *Optimum design as a function of R_0.* Shows how values for key parameters that optimise the precision of a_f (as calculated using Eq.(7)) vary as a function of R_0 for each of the experimental designs in Fig. 2. (a) Single contact group (without dominance) % of seeders. (b) Pure design (without dominance) % of seeders. (c) Pure design (with dominance) % of seeders. (d) Mixed design (without dominance) % of *BB* in group 1 (which is equal to the % of *AA* in group 2). (e) Mixed design (with dominance) % of *BB* in group 1 (which equals the % of *AB*). Other groups have the same proportions but with genotypes permuted.
