# Supplementary material for: Optimal experimental designs for estimating genetic and non-genetic effects underlying infectious disease transmission
Source: Genet Sel Evol. 2022 Sep 5;54:59. doi: 10.1186/s12711-022-00747-1 (PMC9442948; doi:10.1186/s12711-022-00747-1)
Supplement: Supplementary file 13 — Additional file 13: Fig. S10. Design replication. Description: Investigates how the reduction in precision when incorporating group effects can be moderated by means of design replication (that is repeating the same basic designs in Fig. 2 several times). [file 12711_2022_747_MOESM13_ESM.pdf]

Design replication

It was found in Additional file 11 that incorporating group effects substantially reduces the precision of SNP effects. This additional file investigates how this reduction can be moderated by means of design replication (that is repeating the same basic designs in Fig. 2 several times). Figure S10 shows that increasing the number of contact groups N_{group} for a fixed total number of individuals (through replication of the basic design) leads to the SD in SNP effects reducing towards their analytical expectation.

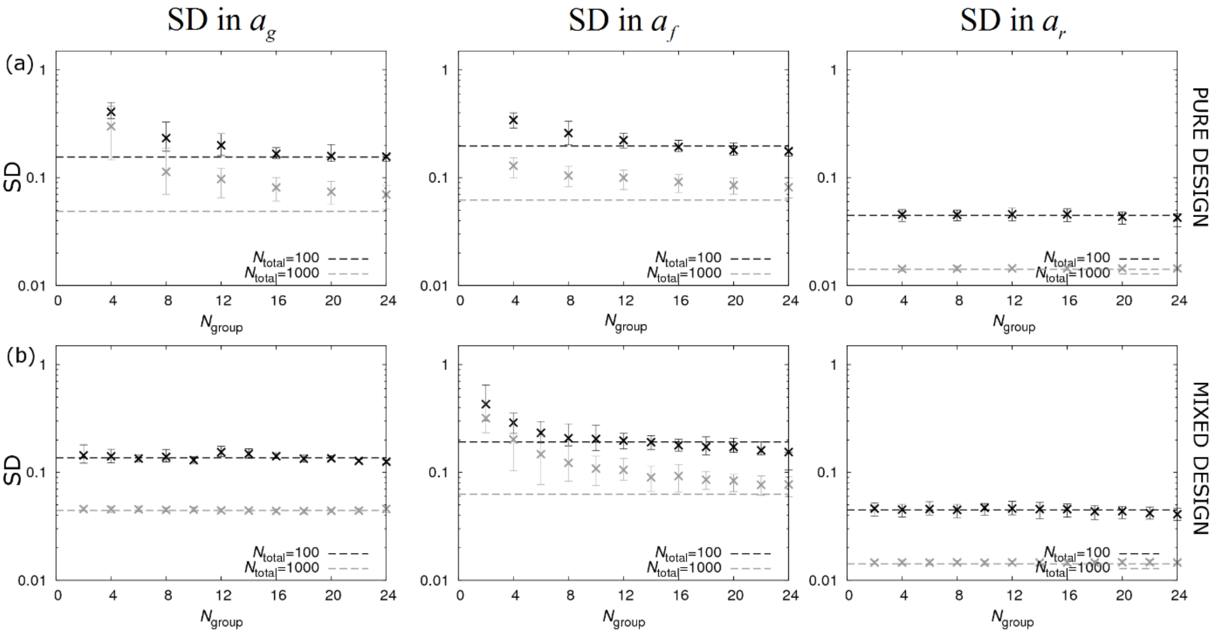

Figure S10. *Reducing the influence of group effects on precision of SNP effect estimates by increasing the number of groups through experimental replicates*. The left, middle and right columns show graphs for standard deviations in the SNP effects for susceptibility a_g, infectivity a_f and recoverability a_r as a function of the number of contact groups (with fixed number of individuals) for a model containing a group effect under optimised (a) pure design without dominance (with 4 contact groups per replicate, see Fig. 2b) and (b) mixed design without dominance (with two contact groups per replicate, see Fig. 2c). Dashed lines represent analytical results and crosses come from posterior estimates from simulated data (see Additional file 1). N_{total} refers to the total number of individuals.
