# Supplementary material for: Optimal experimental designs for estimating genetic and non-genetic effects underlying infectious disease transmission
Source: Genet Sel Evol. 2022 Sep 5;54:59. doi: 10.1186/s12711-022-00747-1 (PMC9442948; doi:10.1186/s12711-022-00747-1)
Supplement: Supplementary file 14 — Additional file 14: Fig. S11. Figure S11 investigates the addition of a single large fixed effect with elements in the design matrix X set in such a way as to give a certain degree of correlation with the SNP. [file 12711_2022_747_MOESM14_ESM.pdf]

Fixed effect correlated with SNP

This additional file investigates adding a single large fixed effect (with size $b_{g0}=b_{f0}=b_{r0}=0.2$) in Eq.(2), with elements in the design matrix $\mathbf{X}$ (which is now a vector because only a single fixed effect is considered) set to values 0 or 1 with equal probability in such a way as to give a certain degree of correlation with the SNP. This correlation is defined by

$$Corr(SNP, X) = \frac{\langle \mathbf{X}, \mathbf{W} \rangle}{\sigma_X \sigma_W}, \tag{A1}$$

where $\mathbf{W}$ is a vector giving the number of A alleles for each individual in the population.

Figure S11 shows results for the pure and mixed designs (without dominance). We find that provided there is little or no correlation between the fixed effect and SNP under investigation, the precision with which parameters can be estimated is largely unaffected. However, if there is significant correlation (especially in the case of inferring a_f) this can result in a significant loss in statistical power due to confounding. Therefore, when designing experiments, care should be taken to randomise (across groups) all factors other than the genotype of the SNP under investigation.

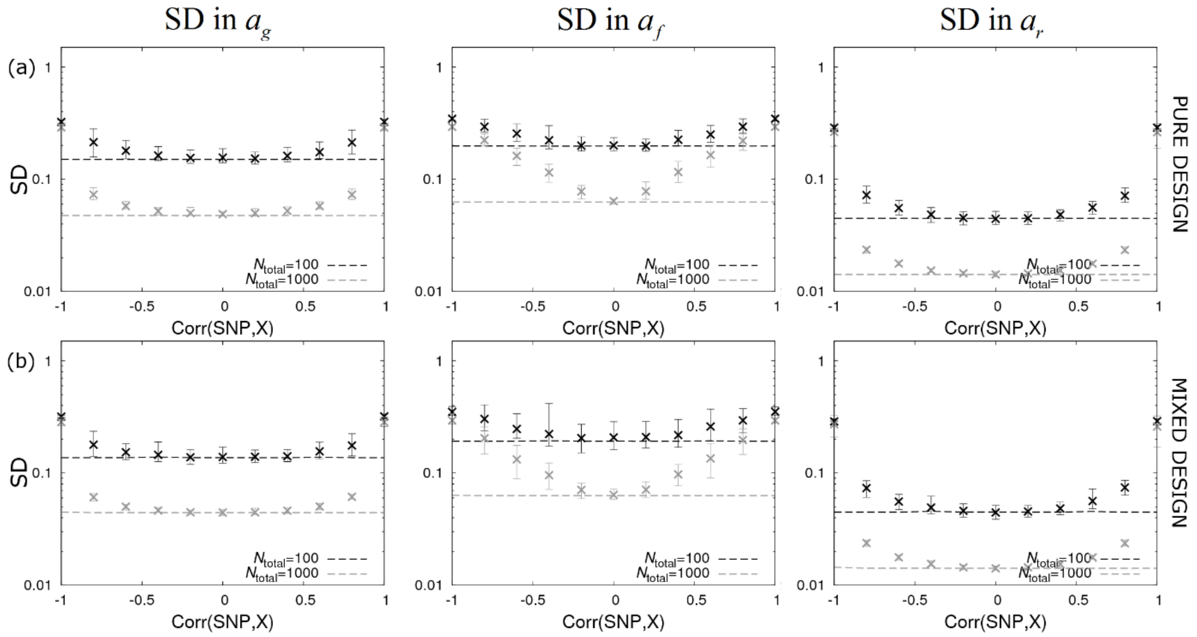

Figure S11. *Impact of a single fixed effect.* The left, middle and right columns show graphs for standard deviations in the SNP effects for susceptibility a_g, infectivity a_f and recoverability a_r as a function of the correlation between the number of A alleles at the SNP (which takes values 0, 1 or 2) and elements of the design matrix $\mathbf{X}$ for a single fixed effect under optimised (a) pure and (b) mixed designs (without dominance). The size of the fixed effect is taken to be $b_{g0}=b_{f0}=b_{r0}=0.2$. Dashed lines represent analytical results and crosses come from posterior estimates from simulated data (see Additional file 1). N_{total} refers to the total number of individuals.
