# Supplementary material for: Optimal experimental designs for estimating genetic and non-genetic effects underlying infectious disease transmission
Source: Genet Sel Evol. 2022 Sep 5;54:59. doi: 10.1186/s12711-022-00747-1 (PMC9442948; doi:10.1186/s12711-022-00747-1)
Supplement: Supplementary file 15 — Additional file 15: Rather than defining the proportions of genotypes in the seeder and contact populations, we consider the case in which individual genotypes are randomly allocated with A allele having frequency p, assuming Hardy-Weinberg equilibrium. Fig. S12. Precision estimates for the SNP parameters with random genotype allocation. [file 12711_2022_747_MOESM15_ESM.pdf]

Genotype determined by Hardy-Weinberg equilibrium

Rather than defining the proportions of genotypes in the seeder and contact populations, here we consider the case in which individual genotypes are randomly allocated with *A* allele frequency p. Assuming the SNP is in Hardy-Weinberg equilibrium and that individuals are unrelated then for a population of size N the following results can be derived:

$$\begin{aligned} \langle \chi \rangle &= 2p-1, \quad \mathrm{Var}(\chi) = \frac{2p(1-p)}{N}, \\ \langle H \rangle &= 1-2p(1-p), \quad \mathrm{Var}(H) = \frac{2p(1-p)\big(1-2p(1-p)\big)}{N}. \end{aligned} \tag{A1}$$

Applying these expression separately to the seeder and contact populations and substituting into the analytical expressions in Eqs.(6)-(8) leads to the curves shown in Fig. S12. Crucially we find that the precision of both the SNP effects and dominance parameters is maximised when the number of seeders N_{seed} is small. In this limit the following results can be derived:

$$\begin{aligned} \text{SD in } a_g &\cong \frac{1}{\sqrt{2p(1-p)N_{\text{total}}}}, \\ \text{SD in } a_f &\cong \frac{1}{\sqrt{2p(1-p)\left(2-\dfrac{1}{G_{\text{size}}-1}\right)N_{\text{group}}}}, \\ \text{SD in } a_r &= \frac{1}{\sqrt{2p(1-p)kN_{\text{total}}}}, \end{aligned} \tag{A2}$$

and for the SDs in the dominance parameters:

$$\begin{aligned} \text{SD in } \Delta_g &\cong \frac{1}{|a_g|\sqrt{2p(1-p)\big(1-2p(1-p)\big)N_{\text{total}}}}, \\ \text{SD in } \Delta_f &\cong \frac{1}{|a_f|\sqrt{4p(1-p)\big(1-2p(1-p)\big)N_{\text{group}}}}, \\ \text{SD in } \Delta_r &= \frac{1}{|a_r|\sqrt{2p(1-p)\big(1-2p(1-p)\big)kN_{\text{total}}}}. \end{aligned} \tag{A3}$$

A key point to note is that the SD in SNP effect for infectivity a_f in Eq.(A2) (and also for the SD in Δ_f in Eq.(A3)) now contains N_{group} in the denominator instead of N_{total}. This means that increasing the number of individuals in contact groups does not substantially increase the precision with which a_f can be estimated (a feature that was noted in [15]).

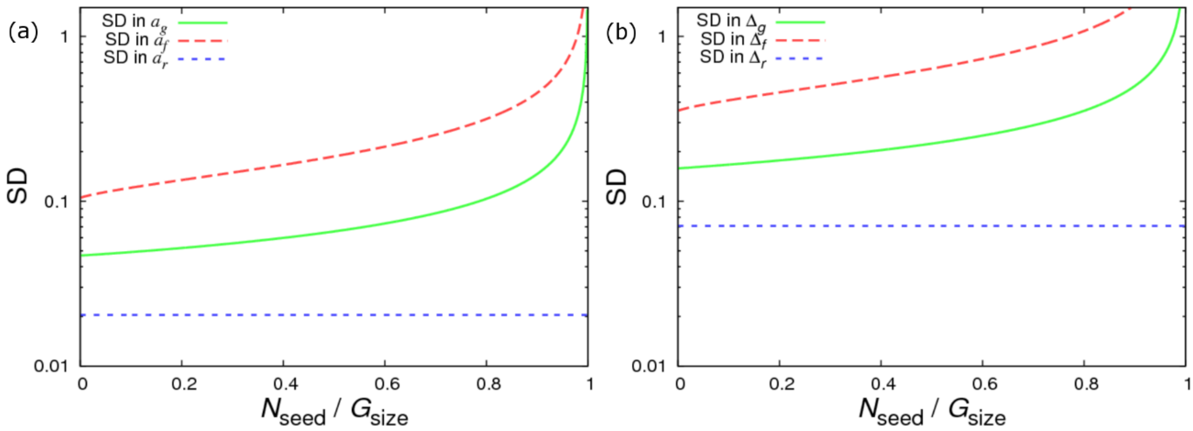

Figure S12. *Precision estimates for the SNP parameters with random genotype allocation.* These results come from the analytical expressions from Eqs.(6-13) (with Eqs.(A2) and (A3) representing the limit of small N_{seed}): (a) SNP effects and (b) dominance parameters. Individual genotypes are randomly sampled assuming Hardy-Weinberg equilibrium with mean *A* allele frequency p=0.4 (over all groups). Furthermore it is assumed that all individuals become infected (*i.e.* ϕ=1) and parameters use were k=5, G_{size}=10 and N_{group}=100.
